# Effects of theta phase precessing optogenetic intervention on hippocampal neuronal reactivation and spatial maps

**DOI:** 10.1016/j.isci.2023.107233

**Published:** 2023-06-28

**Authors:** Yuki Aoki, Taiki Yokoi, Shota Morikawa, Nahoko Kuga, Yuji Ikegaya, Takuya Sasaki

**Affiliations:** 1 Graduate School of Pharmaceutical Sciences, The University of Tokyo, 7-3-1 Hongo, Bunkyo-ku, Tokyo 113-0033, Japan; 2 Department of Pharmacology, Graduate School of Pharmaceutical Sciences, Tohoku University, 6-3 Aramaki-Aoba, Aoba-Ku, Sendai 980-8578, Japan; 3 Institute for AI and Beyond, The University of Tokyo, Tokyo 113-0033, Japan; 4 Center for Information and Neural Networks, 1-4 Yamadaoka, Suita City, Osaka 565-0871, Japan

**Keywords:** Neuroscience, Sensory neuroscience

## Abstract

As animals explore environments, hippocampal place cells sequentially fire at progressively earlier phases of theta oscillations in hippocampal local field potentials. In this study, we evaluated the network-level significance of theta phase-entrained neuronal activity in organizing place cell spike patterns. A closed-loop system was developed in which optogenetic stimulation with a temporal pattern replicating theta phase precession is delivered to hippocampal CA1 neurons when rats traversed a particular region on a linear track. Place cells that had place fields during phase precessing stimulation, but not random phase stimulation, showed stronger reactivation during hippocampal sharp-wave ripples in a subsequent rest period. After the rest period, place cells with place fields that emerged during phase precessing stimulation showed more stable place fields. These results imply that neuronal reactivation and stability of spatial maps are mediated by theta phase precession in the hippocampus.

## Highlights

• Theta phase precessing stimulation is applied to hippocampal CA1 neurons
• Place cells with place fields during stimulation show stronger reactivation
• Place cells with place fields during stimulation show more stable place fields

## Graphical abstract

**Figure:**
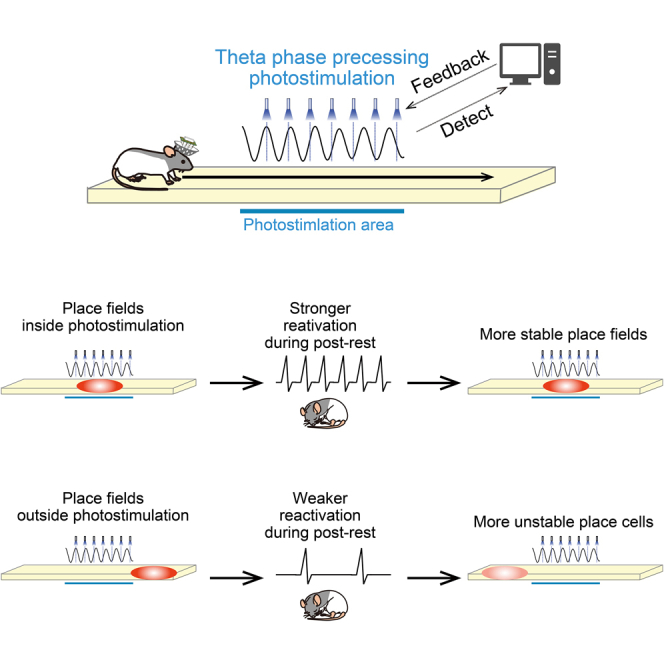

## Introduction

Hippocampal place cells generate spikes when an animal is located in a specific part of the environment^1^; these spikes are considered to constitute a cognitive map in the brain.^2^ The timing of location-specific spikes is tightly associated with theta oscillations in extracellular local field potentials (LFPs).^3^ Importantly, theta phase precession is a unique temporal coding phenomenon in which sequentially occurring place cell spikes gradually shift to earlier phases of the theta cycle (5–10 Hz).^3^^,^^4^^,^^5^

Many studies have demonstrated that repetitive neuronal activation at theta frequencies is optimal to evoke long-term synaptic potentiation in hippocampal neurons both *in vitro*^6^^,^^7^^,^^8^^,^^9^ and *in vivo,*^10^^,^^11^^,^^12^^,^^13^^,^^14^^,^^15^ suggesting that the coordination of place cell firing by theta oscillations effectively exerts plastic changes in the hippocampal circuit and contributes to memory functions. In accordance with these ideas, manipulation of the medial septum in which hippocampal theta power and theta entrainment of neuronal activity are depressed^16^^,^^17^^,^^18^^,^^19^^,^^20^^,^^21^ and lesion of the medial entorhinal cortex in which theta phase precession is partly eliminated^18^ have been shown to cause pronounced impairments in spatial memory.^22^^,^^23^^,^^24^^,^^25^ Moreover, animals showing reduced theta power and phase precession of place cells during passive movement have an impairment in subsequent offline memory replays (∼100 ms),^26^ a memory process thought to be critical for memory consolidation.^27^^,^^28^^,^^29^^,^^30^ Together with the necessity of postexperience reactivation of place cells in the maintenance of place fields,^31^^,^^32^ theta-entrained neuronal activity may be a key mechanism to enhance the stability of hippocampal spatial maps. While these studies with pharmacological and behavioral manipulations have well established the significance of theta-related neuronal activity, a fundamental issue remains concerning whether the observed impairments in spatial memory and memory-related neuronal dynamics were due to an overall reduction in theta power or specifically due to the attenuation of theta phase-related neuronal spikes.

To resolve this technical challenge, we applied a closed-loop system in which hippocampal neuronal populations expressing channelrhodopsin 2 (ChR2) undergo optogenetic stimulation with a temporal pattern mimicking theta phase precession. While similar systems targeting theta phases have been developed in early studies,^33^ this study specifically created a temporal stimulus pattern mimicking theta phase precession. Our study focused exclusively on suprathreshold spike dynamics by multiunit recordings, but this manipulation should essentially evoke subthreshold activity in photostimulated neurons even without apparent spikes; therefore, the activity resulting from this manipulation could potentially replicate and enhance naturally observed theta phase precession of subthreshold voltage oscillations, which underpins the entrainment of suprathreshold spike patterns in theta phase precession.^34^^,^^35^ Pertinently, recent studies have demonstrated that certain subthreshold activity is sufficient to trigger new spatial representations.^36^^,^^37^ On the basis of these insights, we examined how optogenetic intervention that results in phase precession influences neuronal reactivation and spatial representations of hippocampal place cell ensembles.

## Results

### Photostimulation with a temporal pattern mimicking theta phase precession

**Figure 1:**
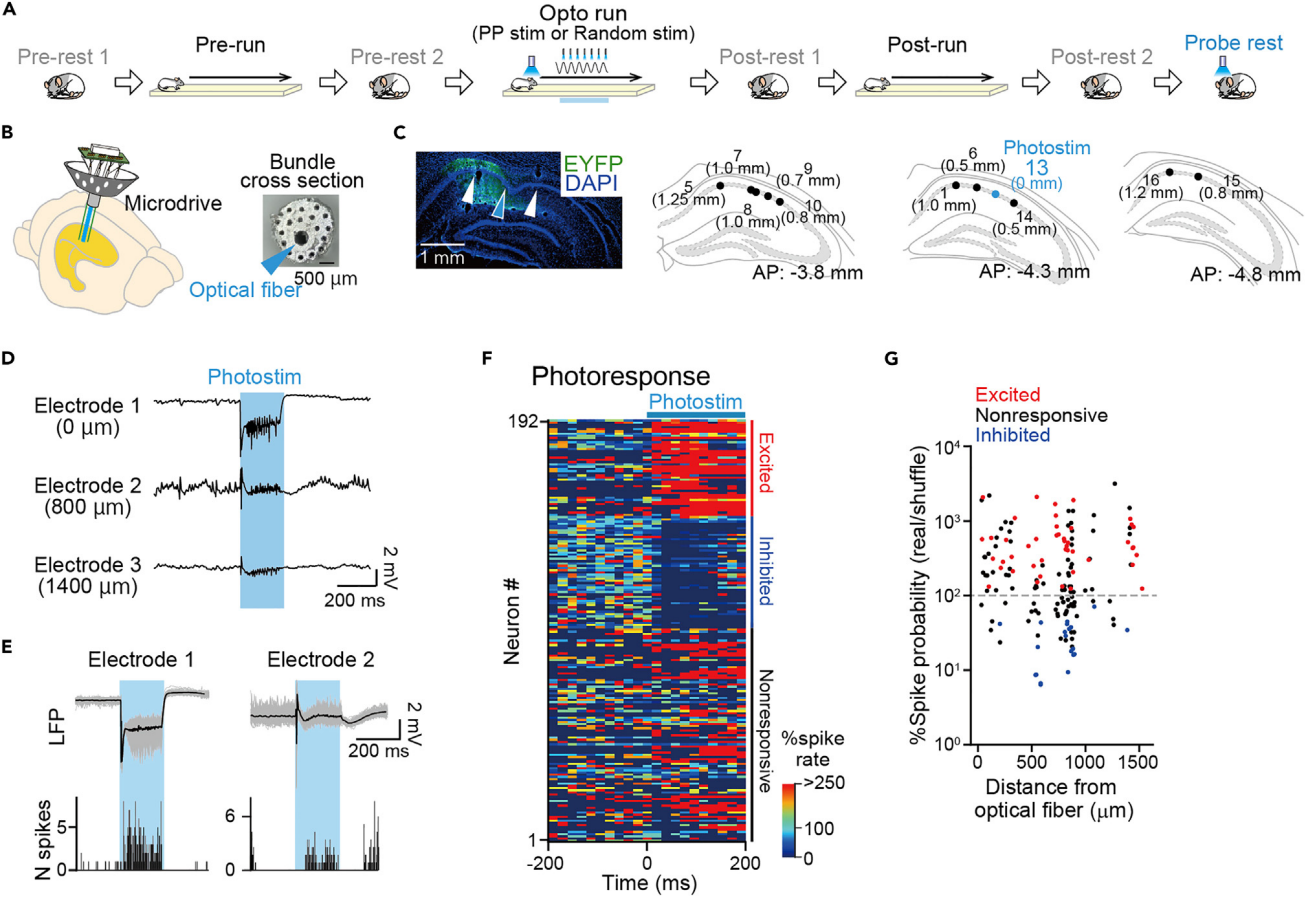
Experimental setup (A) On a recording day, a rat performed three 15-min linear track sessions, flanked by a 60-min rest session. The second run session included photostimulation (opto run). (B) A microdrive with an optical fiber (cyan arrowhead) located at the center of an electrode bundle was implanted into the dorsal hippocampal CA1 region. (C) (Top left) Histological verification of EYFP expression (green) with the locations of tetrode tips (white arrowheads) and an optical fiber (blue arrowhead) superimposed on DAPI-labeled hippocampal neurons (blue). (Bottom and right) Positions of tetrodes (black) and an optical fiber (blue) on the dorsal hippocampal cell layer in sequential coronal brain sections in a rat. The length in parentheses indicates the distance from the optical fiber. (D) LFPs from three representative electrodes in response to 200-ms photostimulation. (E) Corresponding to D, the photostimulation-induced LFPs (top; black thick, average; gray thin, each trial) and spike rate changes in multiunit signals (bottom) in electrodes 1 and 2. (F) Pseudocolor images showing averaged changes in photostimulation-induced spike rates from neurons. The time was aligned to the onset of photostimulation, and the neurons were aligned to cell types based on their photoresponsivity. In each neuron, firing rates in individual bins were normalized by its average firing rate at 0–200 ms before photostimulation. Here, 192 neurons with an average firing rate of more than 0.1 Hz before and after photostimulation were plotted for visualization, whereas the other 132 neurons with an average firing rate of less than 0.1 Hz were not presented. (G) Ratios of photostimulation-induced spike probabilities in real data to those in shuffled data, plotted as a function of distance from the optical fiber. Each dot represents each neuron. Plots were jittered within 100 μm along the horizontal axis for visualization. Cells that did not exhibit photostimulation-induced spikes were excluded from this graph.

Rats were injected with a viral construct, AAV-CaMKII-hChR2(H134R)-EYFP, into the dorsal hippocampal CA1 and CA3 regions, which have been utilized to express ChR2 in excitatory pyramidal neurons in the hippocampus.^38^^,^^39^^,^^40^ Then, independently movable tetrodes and an optical fiber located at the center of the tetrodes were implanted into the hippocampal CA1 region (Figures 1B and S1A). In total, 322 putative excitatory pyramidal cells were recorded from rats with photostimulation (n = 15 total recording days from 6 rats; Table S1). After hippocampal neurons expressed ChR2-EYFP and the tetrodes and the optical fiber reached the hippocampal CA1 region (Figure 1C), recordings with photostimulation were initiated. Under these conditions, photostimulation is considered to induce CA1 neuronal activity in response to both depolarization via direct photostimulation^41^ and synaptic inputs via indirect activation of CA3-CA1 Schaffer collaterals and feedforward/feedback inhibitory networks. On a recording day, rats first rested in a familiar box (pre-rest 1) for 60 min performed a linear track run by running back and forth to obtain chocolate milk rewards placed at both ends (pre-run) for 15 min, rested in the same box (pre-rest 2) for 60 min, performed the same run with photostimulation (opto run) on a photostimulation area for 15 min, rested in the same box (post-rest 1) for 60 min, performed the same run again with no photostimulation (post-run), and rested in the same box (post-rest 2) for 60 min (Figure 1A). In the linear track run, all the rats ran well and completed more than 50 running laps in 15 min; hence, we were able to repeatedly apply photostimulation in the same area and measure neuronal spike responses during running. After post-rest 2, longer photostimulation (light intensity of 3–6 mW, pulse width of hundreds of milliseconds) was repetitively delivered in the same rest box to identify the photoresponsiveness of recorded neurons; this period was termed the probe rest session.

In the probe session, we confirmed that photostimulation induced transient deflections of LFPs and changes in multiunit spiking activity (Figures 1D, 1E, and S1D). No pronounced relationship was observed between electrode-optical fiber distance and the magnitude of photostimulation-induced LFP deflections (Figure S1D). Overall, in the probe rest session, 45/322 pyramidal neurons were identified as excited neurons showing significant increases in spike rates at the time of photostimulation (Figure 1F). In addition, 50/322 neurons were identified as inhibited neurons showing opposite changes in spike rates (Figure 1F), which are likely mediated by inhibitory interneurons.^42^ The other cells were classified as nonresponsive cells. Consistently, a subset of putative inhibitory cells was identified as excited and inhibited neurons (Figure S1E; 2/10 and 4/10 neurons, respectively). No pronounced relationship was observed between electrode-optical fiber distance and photostimulation-induced spike rate changes (Figure 1G). We note that the actual proportions of neurons that received photostimulation-induced effects, including subthreshold depolarization, should be larger than those identified based on the photoinduced spike patterns.^41^

**Figure 2:**
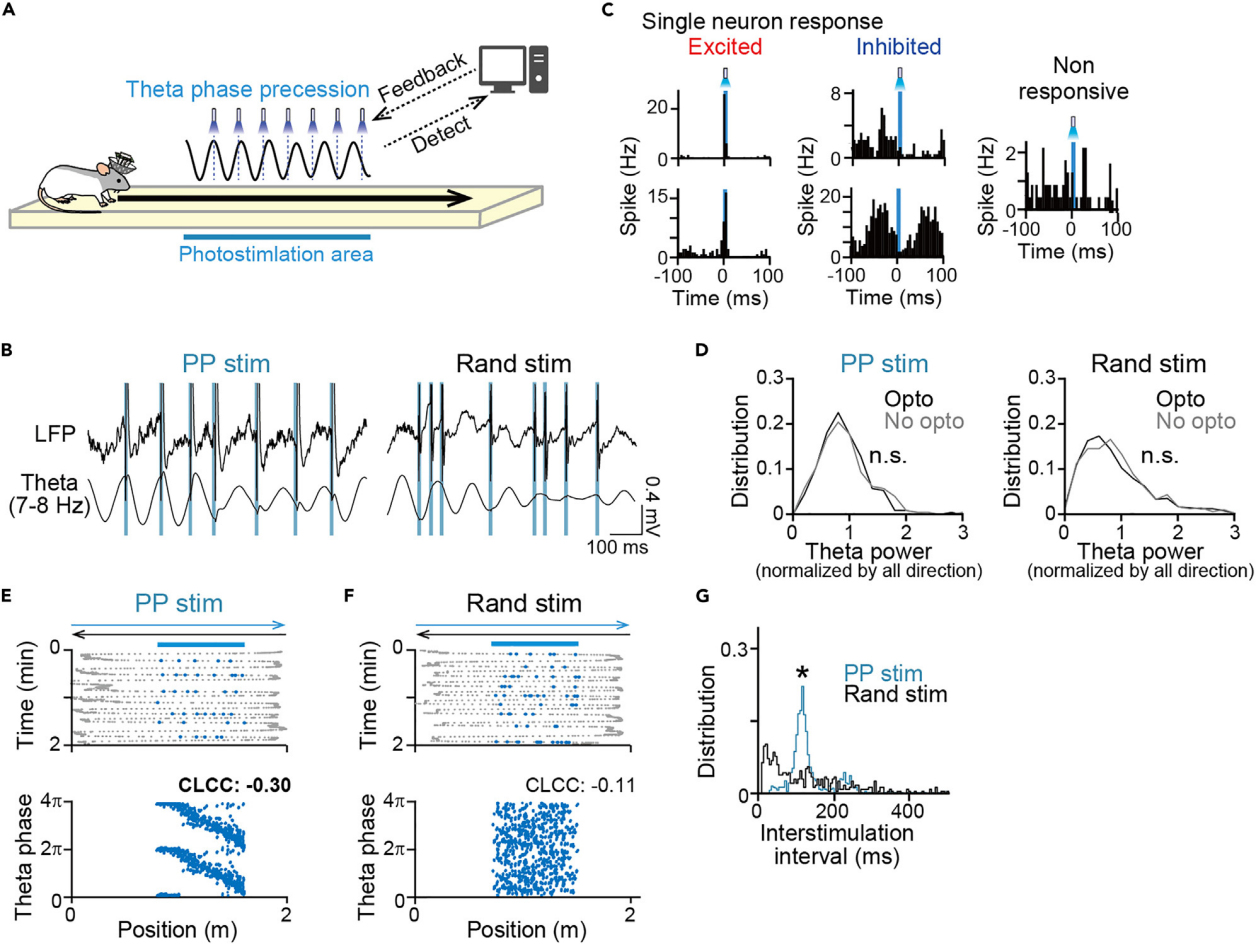
Theta phase precessing optogenetic stimulation into hippocampal CA1 neurons (A) A schematic illustration of the opto run. PP stimulation was applied specifically when a rat traversed through the photostimulation area on a linear track. (B) Original (top) and theta (7–8 Hz) bandpass-filtered (bottom) LFP traces. Blue vertical lines show the timing of photostimulation. (C) Photostimulation-induced spike rate changes aligned to the onset of 5-ms photostimulation in five representative neurons. (D) (Left) Distributions of theta power on the photostimulation area in the opto direction with PP stimulation and the no-opto direction when rats were running with a speed of 50 cm/s in each lap (n = 226 and 225 laps). p > 0.05, Mann-Whitney U test. (Right) Same as the left panel but for random stimulation (n = 472 and 475 laps). (E) (Top) Positions of PP stimulation (blue dots) during running from left to right superimposed on position samples of trajectories of a rat running back and forth on a linear track (gray dots). (Bottom) The theta phase of photostimulation (y axis) represented as a function of position (x axis). CLCC is shown above (significant CLCC highlighted in bold). (F) Same as E but for random phase stimulation. (G) Distributions of interstimulation intervals within a lap between PP stimulation and random phase stimulation. ∗p < 0.05, Mann-Whitney U test.

Under these experimental conditions, photostimulation with single blue light pulses (light intensity of 3–6 mW, pulse width of 5 ms) at hippocampal theta phase precession (PP stimulation) was implemented by a closed-loop feedback system while the rats exhibited sufficient hippocampal theta power and crossed an area of 0.6–1.6 m (photostimulation area) toward one direction (opto direction) (Figures 2A and 2B, left). The 5-ms photostimulation induced transient increases or decreases in spike rates in excited and inhibited neuronal populations (Figure 2C). We confirmed that photostimulation did not alter spike waveforms in individual neurons, compared with those observed from the other run periods and the rest periods without photostimulation (typical neurons shown in Figure S2B). Accordingly, the spike clusters arising from these neurons remained stable over recording periods, including the run, rest, and probe rest periods, enabling continuous tracking of spike clusters of identical neurons (typical clusters shown in Figure S2C). No significant differences in theta power on the photostimulation area (0.6–1.6 m from each start) during running with a speed of more than 50 cm/s were found between the opto direction and the opposite (no opto) direction without PP stimulation (Figure 2D; PP stim: *Z* = 0.27, p = 0.78, Mann-Whitney U test; n = 226 and 225 laps from 5 recordings; random stim: *Z* = 0.57, p = 0.57, Mann-Whitney U test; n = 472 and 475 laps from 10 recordings). These results verified that our closed-loop system preserved overall theta power while selectively inducing intracellular neuronal depolarization and spikes. PP stimulation was applied in all laps in the opto direction (Figure 2E). The mode frequency of photostimulation was 8.8 Hz with an interstimulus interval of 114 ms (Figure 2G), which was slightly higher than the targeted frequency of extracellular theta (7–8 Hz) oscillations,^34^ confirming that this photostimulation frequency represents theta phase precession. The duration per lap in which rats stayed in the photostimulation area was 0.87 ± 0.20 s, and the number of photostimulation pulses per lap was 6.3 ± 2.1 (mean ± standard deviation [SD]).

As a control experiment, we created a random phase stimulation protocol in which photostimulation with similar light intensity and pulse width was randomly delivered irrespective of theta phases (Figure 2B, right, and Figure 2F). In random stimulation protocols, the number of photostimulations per lap in the photostimulation area was set nearly equivalent to that in PP stimulation protocols. The random stimulation protocol had significantly different distributions of interstimulation intervals (Figure 2G; n = 917 and 468; *Z* = 6.50, p = 8.0 × 10^−11^, Mann-Whitney U test), confirming that temporal structures of stimulation were disrupted compared with those of PP stimulation protocols.

### Induction of place fields by PP stimulation

We next evaluated the impact of photostimulation on the spatial representation patterns of place cells. Of a total of 322 putative pyramidal cells analyzed (n = 146 cells from 5 recording days from 3 rats with PP stimulation and n = 176 cells from 10 recording days from 5 rats with random stimulation), 177 (55.0%) place cells were detected in the pre-run session. Of the 177 cells, 60 cells had unidirectional place fields depending on the movement direction, whereas 117 cells had bidirectional place fields observed in both directions. After identifying place cell activity from the pre-run session as a baseline, we examined the effects of PP stimulation on their spatial firing patterns (n = 146 cells from 5 recording days from 3 rats with PP stimulation). The number of laps in the opto run was 99.1 ± 11.7, which was not significantly different from that in the pre-run (n = 5 recording days from 3 rats; *t*_7_ = 0.28, p = 0.78, paired *t* test). Average running speed in the opto direction in the opto run was 57.6 ± 11.6 (n = 244 laps), and the distribution was not significantly different from those in the opposite direction and in the other run sessions (Figure S1F; Tukey’s test, p > 0.05). These results confirm no pronounced effects of photostimulation on behavioral performance. While a recent study has reported that activation of behavior-relevant place cells alter memory-based spatial behavior,^43^ our results demonstrated that the rats running on the linear track showed no pronounced changes in their behavioral patterns in response to photostimulation, possibly because this running behavior alone is less dependent on spatial memory components.

**Figure 3:**
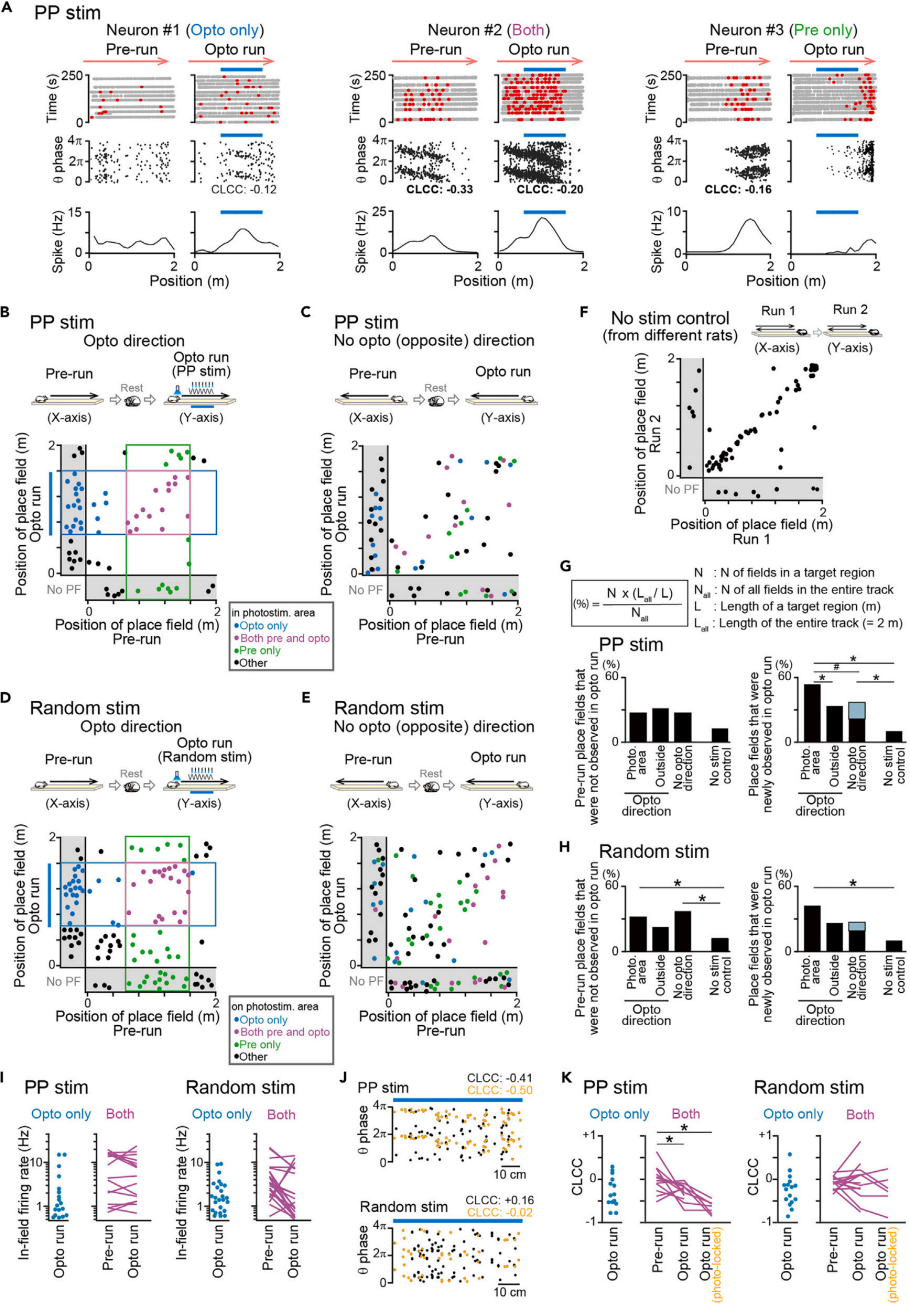
Place fields in the opto run (A) Typical spatial spike patterns of neurons with a place field on the photostimulation area in the opto run only (left), place fields on the photostimulation area in both the pre-run and opto run (middle), and a place field on the photostimulation area in the pre-run only (right). (Top) Trajectories (gray) with the locations of individual spikes (red). (Middle) The theta phase of spikes as a function of position (two theta cycles). CLCC is shown below for each place field defined in the photostimulation area (significant CLCC highlighted in bold). (Bottom) Spatial firing-rate map. The upper cyan bars represent the photostimulation area. The other examples are shown in Figure S3A. (B) Joint plots showing changes in the positions of the place field center between the pre-run (x axis) and the opto run with PP stimulation (y axis) (n = 72 cells that had at least one place field in the two sessions). Each dot represents each cell, and each cell is labeled in a different color depending on the types of place fields. Plots were jittered within 5 cm for visualization. (C) Same as B but for the opposite (no-opto) direction (n = 73 cells that had at least one place field in the two sessions). Each cell is labeled in a different color according to the classification in B. Plots were jittered within 5 cm for visualization. (D and E) Same as B and C but for random phase stimulation (D, n = 112 cells; E, 105 cells). Plots were jittered within 5 cm for visualization. (F) Data were obtained from three control rats without any stimulation (for detailed protocols, see Figure S7A). Both directions were combined in the joint plot (n = 69 fields that were recorded in at least one run). Plots were jittered within 5 cm for visualization. (G) (Top inset) Calculation of proportions from the plots in B–F. (Left) The proportions of place fields that were observed in the pre-run but not in the opto run with PP stimulation (photo. area and outside from B; no-opto direction from C; no stim control from F; *N*_*all*_ = 46, 46, 51, and 63 cells in total). (Right) The proportions of place fields that were newly observed in the opto run with PP stimulation (*N*_*all*_ = 59, 59, 59, and 61 cells). The thin blue area in the no-opto direction represents a subset of cells (n = 9 cells) that exhibited new place fields in the photostimulation area shown in the leftmost column. ∗p < 0.05, chi-squared test followed by Bonferroni correction. ^#^p < 0.05, chi-squared test when the cells with new place fields in the photostimualtion area were removed. (H) Same as G but for random phase stimulation (left, *N*_*all*_ = 81, 81, 85, and 63 cells; right, *N*_*all*_ = 90, 90, 73, and 61 cells in total). (I) (Left) In PP stimulation, in-field firing rates of neurons with a place field on the photostimulation area in the opto run only (left dots: n = 20 cells) and comparisons of in-field firing rates of neurons with place fields on the photostimulation area in both the pre-run and opto run between the pre-run and opto run (right lines: n = 15 cells). Each dot and line show each cell. (Right) Same as the left panel but for random phase stimulation (left dots: n = 25 cells; right lines; n = 21 cells). (J) Examples of the theta phase of spikes as a function of position (two theta cycles) in the poststimulation area for a cell for PP stimulation (top) and random phase stimulation (bottom). Data from all laps were superimposed. Black and orange dots show all spikes and photostimulation-locked spikes, respectively. CLCC computed from each condition is shown above. (K) CLCC of neurons with a place field on the photostimulation area in the opto run only (left dots; n = 14 and 16 cells) and comparisons of CLCC of neurons with place fields on the photostimulation area in both the pre-run and opto run among the pre-run, opto run, and the opto run with poststimulation-locked spikes only (right lines; n = 12, 13, 7 and 18, 15, 6 cells). Each dot and line show each cell. ∗p < 0.05, Mann-Whitney U test followed by Bonferroni correction.

Figure 3A shows three typical cells showing place-selective firing in the pre-run and opto run with PP stimulation, and Figure 3B shows a joint plot summarizing the positions of all place fields observed in the pre-run and opto run. To remove the effects of stop behavior and reward on spatial firing patterns, place fields defined at the end of the track were excluded from all analyses. We confirmed that the proportions of place fields in an area corresponding with the photostimulation area and outside the area in the pre-run before PP stimulation did not significantly differ across the opto direction, the no-opto (opposite) direction, and control conditions (Figure S3B; p > 0.05, chi-squared test followed by Bonferroni correction), demonstrating no initial sampling biases of spatial distributions of place fields in the rats with PP stimulation.

To evaluate the effects of photostimulation on changes in place fields, we first computed the proportions of place fields that were observed in the photostimulation area with PP stimulation in the pre-run but not observed in the track in the opto run (Figure 3G, left; n = 6 cells) and the proportions of place fields that were not observed in the track in the pre-run but newly observed in the photostimulation area in the opto run (Figure 3G, right; n = 16 cells). For comparisons, the same analyses were applied to place fields outside the photostimulation area in the opto direction (outside; n = 7 and 10 cells, respectively) and place fields in the no-opto direction (no-opto direction; n = 14 and 22 cells). Furthermore, the same analyses were applied to place fields recorded from different control rats performing the same run with no photostimulation, termed no-stimulation control (Figures 3F and 3G; the detailed protocol is shown in Figure S7A; n = 72 cells recorded from 3 recording days from 3 rats in total; no stim control; n = 8 fields out of 63 fields and 6 fields out of out of 61 fields). Among these four groups, no significant differences in the proportions of place fields that were not observed in the next (opto or control) run were found (Figure 3G, left; p > 0.05, chi-squared test followed by Bonferroni correction). On the other hand, the proportion of place fields that were newly observed in the photostimulation area was significantly higher than those outside the photostimulation area (Figure 3G, right; χ^2^ = 6.80, p = 0.027, chi-squared test followed by Bonferroni correction) and in the no-stimulation control (χ^2^ = 27.32, p = 5.1 × 10^−7^), implying a pronounced increase in new place fields in the photostimulation area. However, no significance was detected when the proportions were compared between the photostimulation area and the no-opto direction (indicated by # in Figure 3G, right; χ^2^ = 3.41, p = 0.063, chi-squared test). The proportion in the no-opto direction was significantly higher than that in the no-stimulation control (χ^2^ = 12.63, p = 0.0011, chi-squared test followed by Bonferroni correction). When we scrutinized cells contained in this unexpectedly higher proportion in the no-opto direction, we noticed that the proportion represented a subset of the cells (n = 9 cells) that showed new place fields in the photostimulation area (represented as thin blue area in the no-opto direction in Figure 3G, right). Consistent with this idea, the cells with new place fields in the photostimulation area showed a significantly higher probability to develop new place fields in the no-opto direction as well, compared with the other cells (χ^2^ = 4.44, p = 0.035, chi-squared test). This result suggests that cells that newly acquired place fields by PP stimulation more likely generate new place fields in the opposite direction as well, possibly owing to their increased intrinsic excitability. When these cells were removed from a statistical analysis (corresponding with the black area only in the no-opto direction in Figure 3G, right), a significance was detected in the comparison between the photostimulation area and the no-opto direction (χ^2^ = 8.29, p = 0.0085, chi-squared test; indicated by # in Figure 3G, right). Taken all, these results confirm that PP photostimulation effectively increases new place fields in the photostimulation area, compared with the other areas and control conditions.

The same analyses were performed for random phase stimulation (Figures 3D–3F and 3H; n = 176 cells from 10 recording days from 5 rats with random stimulation). The proportions of place fields that were observed in the photostimulation area (n = 13 cells; χ^2^ = 7.40, p = 0.020, chi-squared test followed by Bonferroni correction) and the opposite direction (n = 32 cells; χ^2^ = 11.42, p = 0.0022) in the pre-run but not in the opto run with random stimulation were significantly higher than those in the no-stimulation control (Figure 3H, left), implying that random phase stimulation reduced place fields. However, we note that this effect is partly explained by the fact that place field distributions were initially biased to the photostimulation area in the pre-run before random stimulation, compared with no-stimulation control (Figure S3B; χ^2^ = 9.15, p = 0.025, chi-squared test followed by Bonferroni correction). The proportion of place fields that were newly observed in the photostimulation area was significantly higher than that in the no-stimulation control (χ^2^ = 18.47, p = 5.2 × 10^−5^), confirming that random phase photostimulation increases new place fields on the photostimulation area, similar to PP photostimulation. Taken all, these results demonstrate that both PP photostimulation and random phase stimulation are effective to activate certain cell populations to create, at least apparently, new place fields on the stimulation area.

Based on the locations of the photostimulation area and place fields, we further classified three major types of place fields: (1) place fields on the photostimulation area in the opto run only (Figure 3A, neuron #1, opto only; represented in the cyan area in Figures 3B and 3D), (2) place fields on the photostimulation area in both the pre-run and opto run (Figure 3A, neuron #2, both pre and opto; represented in the magenta areas in Figures 3B and 3D), and (3) place fields on the photostimulation area in the pre-run only (Figure 3A, neuron #3, pre only; represented in the green areas in Figures 3B and 3D). The proportions of place fields on the photostimulation area in the opto run only and place fields on the photostimulation area in both the pre-run and opto run were larger in excited neurons than in inhibited neurons and nonresponsive neurons (Figure S3C). This categorization based on apparent locations of place fields was mainly utilized in following analyses.

In neurons with place fields on the photostimulation area in both the pre-run and opto run, no significant differences in in-field firing rates in the photostimulation area were found between the pre-run and opto run during both PP stimulation and random phase stimulation (Figure 3I, both,; PP stim: n = 15 cells, *t*_14_ = 0.56, p = 0.58; random stim: n = 21 cells, *t*_20_ = 1.57, p = 0.13, paired *t* test). In neurons with place fields on the photostimulation area in the opto run only, no significant differences in their in-field firing rates in the photostimulation area were found between PP stimulation and random phase stimulation (Figure 3I, opto only; n = 20 and 25 cells, *Z* = 0.51, p = 0.61, Mann-Whitney U test). These results suggest that there are no overall pronounced differences in stimulation-induced in-field firing rate changes between PP stimulation and random phase stimulation. To evaluate the degree of phase precession of these spike patterns in the photostimulation area, a circular-linear correlation coefficient (CLCC) for each neuron was computed from a position-phase plot consisting of all spikes observed in the photostimulation area (Figure 3J, shown in black) or specifically from spikes that were temporally locked to photostimulation (shown in orange). In neurons with place fields on the photostimulation area in both the pre-run and opto run, CLCC computed from both spike patterns was significantly lowered in the opto run with PP stimulation, compared with the pre-run (Figure 3K, left; all spikes: n = 13 cells, *Z* = 2.48, p = 0.027; photostimulation-locked spikes: n = 7 cells, *Z* = 3.42, p = 0.0024, Mann-Whitney U test followed by Bonferroni correction). Such significance was not observed in the opto run with random phase stimulation (Figure 3K, right; all spikes: n = 15 cells, *Z* = 0.24, p > 0.99; photostimulation-locked spikes: n = 6 cells, *Z* = 1.50, p = 0.27, Mann-Whitney U test followed by Bonferroni correction). These results confirm that PP stimulation is more effective to induce theta phase precession in these neuronal populations. In neurons with place fields on the photostimulation area in the opto run only, no significant differences in CLCC were found between PP stimulation and random phase stimulation (Figure 3K; n = 14 and 16 cells, *Z* = 0.018, p = 0.99, Mann-Whitney U test). In addition, the CLCC in both types of stimulation was not significantly different from 0 (the null hypothesis) (PP stim: *t*_*13*_ = 0.81, p = 0.43; random stim: *t*_*15*_ = 0.74, p = 0.47, *t test* versus 0).

### Increased post-experience spike rates of place cells by PP stimulation

**Figure 4:**
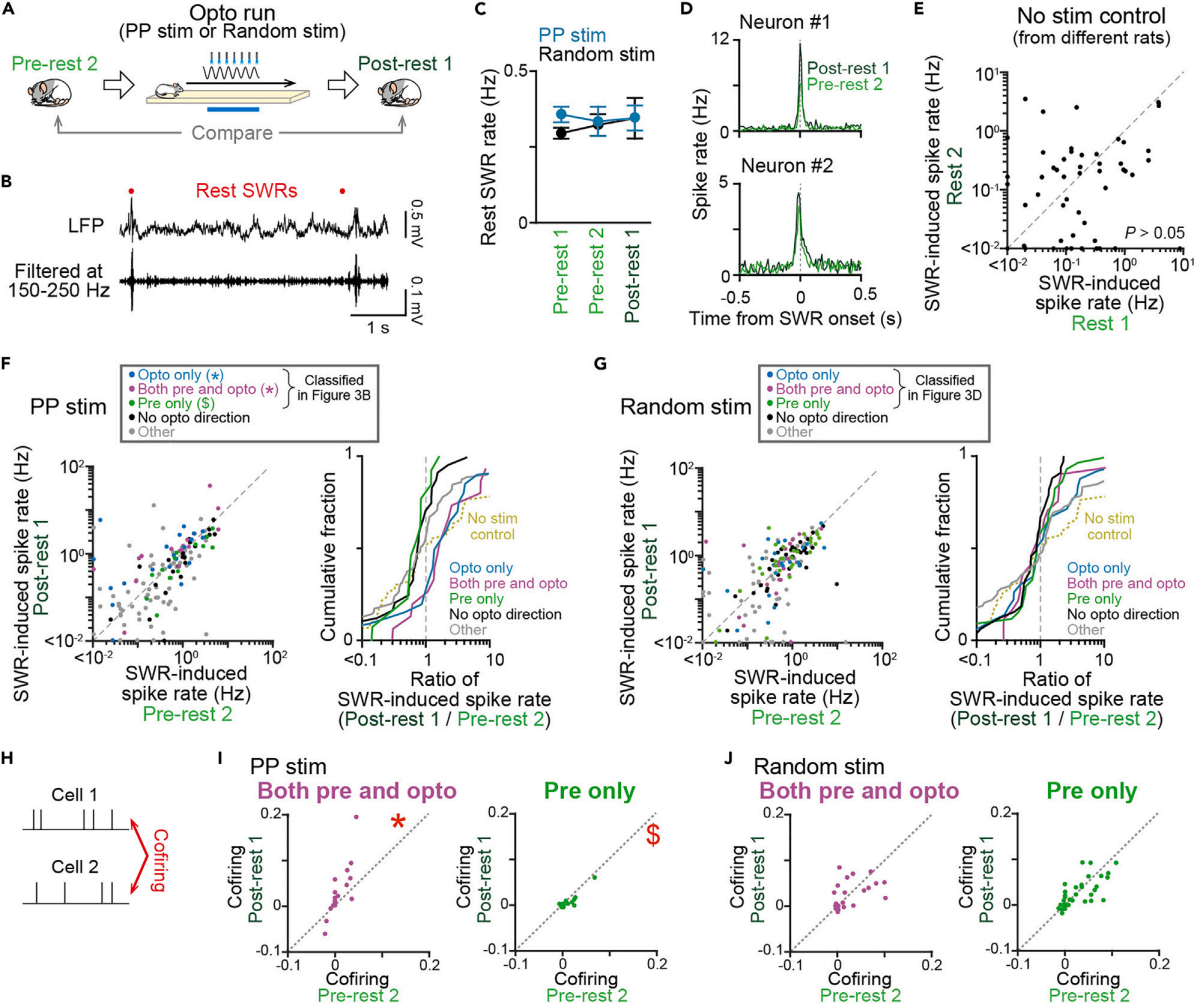
Post-experience spikes of place cells in the subsequent rest period (A) Rest periods before and after the opto run, termed pre-rest 2 and post-rest 1 periods, respectively, were compared. (B) Unfiltered and bandpass-filtered (150–250 Hz) LFP traces of typical SWRs, indicated by red dots. (C) The event rates of SWRs in the rest periods. p > 0.05, paired *t test* followed by Bonferroni correction. Data are represented as mean ± SEM. (D) SWR-triggered spike rate changes in pre-rest 2 and post-rest 1 periods. (E) Comparison of SWR-induced spike rates of individual cells between rest 1 and rest 2 periods (before and after the run 2, respectively) in control mice (n = 72 cells). (F) (Left) Comparison of SWR-induced spike rates of individual cells between pre-rest 2 and post-rest 1 periods. Each dot represents each cell labeled in different colors depending on their place field types classified in Figures 3B and 3C (n = 20, 15, 13, 24, and 74 cells recorded in PP stimulation protocols), and cells were labeled in different colors according to the classification in Figure 3B. ∗ and $ represent significantly higher and lower spike rates in the post-rest 1 period, respectively, compared with the pre-rest 2 period (p < 0.05, Wilcoxon signed-rank test). (Right) Cumulative distributions of percentage changes in SWR-induced spike rates between post-rest 1 and pre-rest 2 periods. For comparison, data from the no-stimulation control group shown in E are represented by the yellow line. (G) Same as F but for random phase stimulation (n = 25, 21, 29, 34, and 67 cells recorded in random stimulation protocols). No significant differences were found in all groups (p > 0.05). (H) For a pair of neurons, a cofiring was computed as a correlation coefficient of their spike counts (bin = 50 ms). (I) (Left) Comparison of cofiring of pairs of neurons with place fields on the photostimulation area in both the pre-run and opto run between pre-rest 2 and post-rest 1 periods. Each dot represents each cell pair (n = 19 cell pairs recorded in PP stimulation protocols). ∗ represents significantly higher cofiring in the post-rest 1 period, compared with the pre-rest 2 period (p < 0.05, Wilcoxon signed-rank test). (Right) Same as the left panel but for pairs of neurons with place field on the photostimulation area in the pre-run only. $ represents significantly lower cofiring in the post-rest 1 period, compared with the pre-rest 2 period (n = 15 cell pairs recorded in PP stimulation protocols; p < 0.05, Wilcoxon signed-rank test). (J) Same as I but for random phase stimulation (n = 18 and 28 cell pairs recorded in random phase stimulation protocols; p > 0.05, Wilcoxon signed-rank test).

We next asked how neuronal populations with these various types of place fields related to photostimulation were reactivated in a subsequent rest period (Figure 4A). Sharp wave ripples (SWRs) were detected from the 60-min rest periods between the run sessions (Figure 4B). The frequency of SWR rates did not significantly differ before (pre-rest 1 and 2) and after (post-rest 1) the opto run in either of the photostimulation protocols (Figure 4C; p > 0.05 for all comparisons, paired *t test* followed by Bonferroni correction). To test how these SWRs reactivated hippocampal neurons, we compared SWR-triggered neuronal spike rates (from 50 ms before to 50 ms after SWR onset) between pre-rest 2 and post-rest 1 periods (two representative neurons shown in Figure 4D and all neurons shown in Figures 4F and 4G). In the no-stimulation control group, no significant differences were found between two rest (rest 1 and rest 2) periods before and after the run 2 period (Figure 4E; n = 72 cells, *Z* = 1.58, p = 0.11, Wilcoxon signed-rank test). In PP stimulation protocols, significantly higher SWR-triggered spike rates in the post-rest 1 period, compared with the pre-rest 2 period were observed from neurons with place fields on the photostimulation area in the opto run only (represented by ∗ in Figure 4F, left; n = 20 cells, *Z* = 2.43, p = 0.015, Wilcoxon signed-rank test; Figure 4F right summarizes the change ratios of SWR-induced spike rates in the post-rest 1 period to those in the pre-rest 2 period) and neurons with place fields on the photostimulation area in both the pre-run and opto run (n = 15 cells, *Z* = 2.33, p = 0.018). Because PP photostimualtion changed in-field firing rates of a subset of neurons with place fields on the photostimulation area in both the pre-run and opto run (Figure 3I, right), the significant increase in SWR-related spikes might be caused by changes in overall cell excitability during a run period, as shown by previous studies.^44^^,^^45^ To control this effect, we randomly downsampled spikes in a rest period for each neuron so that changes in their average spike rates between the two rest periods were normalized by a fold change of in-field spike rates observed from the pre-run to the opto run (Figure S4A). These downsampled datasets similarly showed higher SWR-triggered spike rates in the post-rest 1 period, compared with the pre-rest 2 period (Figure S4B; n = 15 cells, *Z* = 2.16, p = 0.030, Wilcoxon signed-rank test). This result confirms that photostimulation-induced in-field spike rate changes alone cannot account for the increased SWR-related spikes and suggests that the induction of phase precession by stimulation (Figure 3K, left), rather than simple changes in spike rates, contributes to increases in SWR-triggered spikes of neurons with place fields on the photostimulation area in both the pre-run and opto run. In contrast, neurons with place fields on the photostimulation area in the pre-run only showed significantly lowered SWR-triggered spike rates in the post-rest 1 period (represented by $ in Figure 4F, left; n = 13 cells, *Z* = 2.41, p = 0.013, Wilcoxon signed-rank test). These results demonstrated that hippocampal neurons that newly acquired or continuously retained their place fields during PP photostimulation showed increased reactivation in hippocampal SWRs, whereas neurons that lost their place fields during PP photostimulation showed decreased reactivation. No significant differences were observed in neurons that were not classified into these three groups but had place fields in the no-opto direction (Figure 4F; no-opto direction, n = 24 cells, *Z* = 1.51, p = 0.13) and the other neurons (other, n = 74 cells, *Z* = 1.16, p = 0.25).

To further test whether these changes in SWR-related reactivation were specifically observed in cell types defined from the opto run, we classified neurons based on their place fields relative to a pseudo photostimulation area (a region in which photostimulation was not actually present but the relative location on a track was similar to that of the photostimulation area in the opto run) in the no-opto direction (Figure S5A) or in the rats with no-stimulation control (Figure S5E). These place fields were termed pseudo-opto only, pseudo-both, pseudo-pre only, and pseudo-other fields. All neuron types classified based on these place fields both in the no-opto direction and in the rats with no-stimulation control showed no significant differences in SWR-triggered spike rates between the pre-rest 2 and the post-rest 1 periods (Figures S5B and 5F; p > 0.05 in all field types tested, Wilcoxon signed-rank test). These results further confirm that the observed changes in SWR-related reactivation were specific to neuron types with place fields related to photostimulation during the opto run, but not the other runs without photostimulation. In random phase stimulation, no significant differences in SWR-triggered spike rates were observed between the pre-rest 2 and post-rest 1 periods in all cell types tested (Figure 4G; p > 0.05 in all groups, Wilcoxon signed-rank test).

In addition to the results at the level of single neurons, we further examined whether the spikes of photostimulation-related place cells in the rest periods synchronously occurred at the level of neuronal interactions, considered as a crucial neuronal substrate for memory consolidation.^46^^,^^47^ For each neuronal pair, the degree of neuronal synchronization was quantified as cofiring, a correlation coefficient of their spike counts with a bin of 50 ms^46^^,^^47^ (Figures 4H and S6A). In PP stimulation protocols, pairs of neurons with place fields on the photostimulation area in both the pre-run and opto run showed significantly higher cofiring in the post-rest 1 period, compared with the pre-rest 2 period (represented by ∗ in Figure 4I, left, and Figure S6B; n = 19 cell pairs, *Z* = 2.33, p = 0.020, Wilcoxon signed-rank test), demonstrating increased synchronized firing after PP stimulation in the opto run. In contrast, pairs of neurons with place fields on the photostimulation area in the pre-run only showed significantly lower cofiring (represented by $ in Figure 4I, right; n = 15 cell pairs, p = 0.0087), demonstrating decreased synchronized firing. In random phase stimulation protocols, all neuron pairs showed no significant changes in cofiring (Figures 4J and S6C; p > 0.05, Wilcoxon signed-rank test by Bonferroni correction). These results suggest that neuronal ensembles with place fields on the photostimulation area with PP stimulation, but not random phase stimulation, specifically increased their synchronized spikes in a subsequent rest period.

### Increased stability of spatial maps by PP stimulation

**Figure 5:**
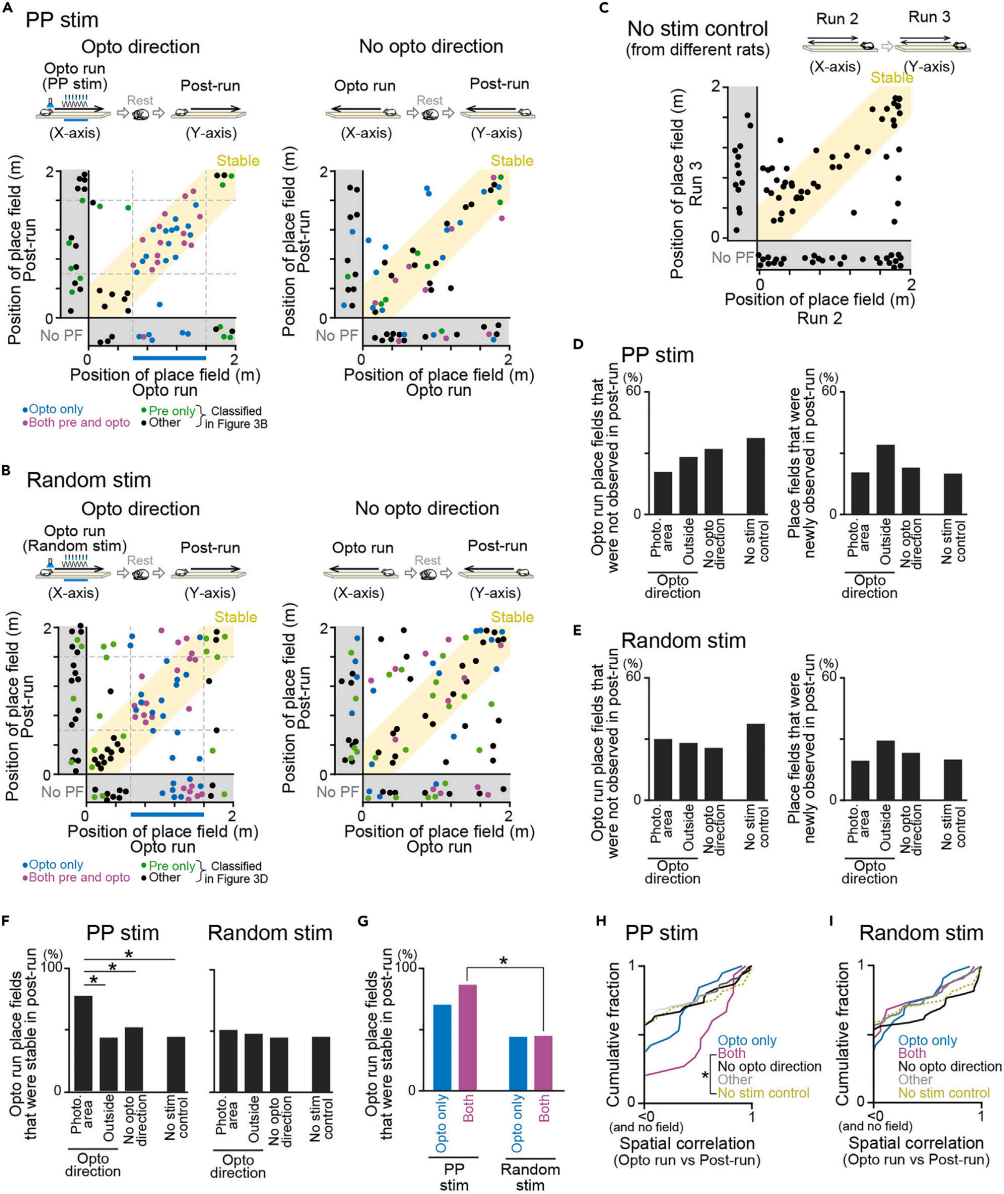
Stability of place fields in the post-run (A) (Left) Joint plots showing changes in place field locations between the opto run (x axis) and the post-run (y axis) (n = 72 cells). Each cell is labeled in a different color according to the classification in Figure 3B. The right panel shows results from the no-opto direction (n = 70 cells). The yellow region represents stable place fields that shifted their field center with a distance of less than 30 cm across the two sessions. Plots were jittered within 5 cm for visualization. (B) Same as A but for random phase stimulation (n = 106 and 90 cells). Plots were jittered within 5 cm for visualization. (C) Same as A but for the no-stimulation control group in which two run periods were compared (n = 87 cells). Plots were jittered within 5 cm for visualization. (D) (Left) The proportions of place fields that were not observed in the post-run after PP stimulation (photo. area and outside from A; no-opto direction from B; no stim control from C; *N*_*all*_ = 56, 56, 58, and 74 cells). (Right) The proportions of place fields that were newly observed in the post-run (*N*_*all*_ = 58, 58, 51, and 58 cells). p > 0.05, chi-squared test followed by Bonferroni correction. (E) Same as D but for random phase stimulation (left, *N*_*all*_ = 86, 86, 73, and 74 cells; right, *N*_*all*_ = 81, 81, 72, and 58 cells in total). (F) (Left) The proportions of place fields that were stable in the post-run after PP stimulation (photo. area and outside from A; no-opto direction from B; no stim control from C; n = 31, 21, 52 and 74 cells). (Right) Same as the left panel but for random phase stimulation (n = 42, 42, 72, and 74 cells). ∗p < 0.05, chi-squared test followed by Bonferroni correction. (G) The proportions in the photostimulation area in F were separately for two types of place fields (opto only: n = 20 and 25 cells; both: n = 15 and 21 cells). ∗p < 0.05, chi-squared test followed by Bonferroni correction. (H) Cumulative distributions of spatial correlations between the opto run and post-run. Correlations from cells with no fields observed in either one of the sessions were computed as 0. ∗p < 0.05, Mann-Whitney U test. (I) Same as H but for random phase stimulation.

Next, we examined how the place fields were altered in the post-run. Joint plots were constructed to compare the positions of place fields between the opto run with PP stimulation and the post-run (Figure 5A; n = 72 and 70 cells). The cells were labeled in different colors based on the classification from the comparisons between the pre-run and opto run in Figure 3B. The same plots were created for random phase stimulation (Figure 5B; n = 106 and 90 cells) and no-stimulation control (Figure 5C; n = 87 cells). No significant differences in spatial information of individual place fields were observed between the pre-run and the post-run (Figure S3D; p > 0.05, Mann-Whitney U test in all comparisons), demonstrating that the quality of place fields was not altered by photostimulation. Similar to Figures 3G and 3H, we computed the proportions of place fields that were not observed or newly observed in the post-run, compared with the opto run (Figures 5D and 5E). In both PP stimulation and random phase stimulation protocols, no significant differences in these proportions were found across the four groups: photostimulation area, outside, no-opto direction, and no-stimulation control (p > 0.05, chi-squared test followed by Bonferroni correction).

Next, to quantify how neurons in the post-run retained their place fields at similar locations to the opto run, we computed the proportions of stable place fields across the opto run and the post-run (yellow regions in the joint plots in Figures 5A–5C). The proportion of place fields that were stable in the photostimulation area with PP stimulation was significantly higher than those outside the photostimulation area (Figure 5F, left; χ^2^ = 6.86, p = 0.027, chi-squared test followed by Bonferroni correction), in the no-opto direction (χ^2^ = 5.77, p = 0.049), and in the no-stimulation control (χ^2^ = 10.14, p = 0.0044). Such significance was not observed in place fields classified relative to the pseudo photostimulation area in both the no-opto direction (Figure S5C) and the no-stimulation control groups (Figure S5G) (p > 0.05 in all field types tested). These results demonstrate that place fields in the photostimulation area were more stably observed thereafter when the stimulation was no longer applied, compared with the other areas and conditions. On the other hand, random phase stimulation did not show such significance (Figure 5F, right; p > 0.05, chi-squared test followed by Bonferroni correction), suggesting no pronounced effects of random phase stimulation on the stabilization of spatial representations.

By further evaluating which types of fields were stable, we found that neurons with place fields on the photostimulation area in both the pre-run and opto run showed a significantly higher proportion of stable place fields in PP stimulation than in random phase stimulation (Figure 5G; n = 15 and 21 cells, χ^2^ = 5.78, p = 0.032, chi-squared test followed by Bonferroni correction). Such significance was not observed from neurons with place fields on the photostimulation area in the opto run only (n = 20 and 25 cells, χ^2^ = 3.04, p = 0.16). These results suggest that place fields that were present before PP stimulation are more stably observed at the identical locations. To further evaluate the stability, spatial correlations were computed from spatial firing-rate distributions between the opto run and post-run for individual place fields detected in the opto run with PP stimulation. Overall, photostimulation-related place fields showed significantly positive spatial correlations (Figure 5H; p < 0.05, Mann-Whitney U test versus 0 within each group), verifying that these place fields are stably represented. In addition, neurons with place fields on the photostimulation area in both the pre-run and opto run showed significantly higher correlations than those in the no-stimulation control groups (Figure 5H; *Z* = 2.03, p = 0.043, Mann-Whitney U test). Such significance was not observed in place fields classified relative to the pseudo photostimulation area in both the no-opto direction (Figure S5D) and the no-stimulation control groups (Figure S5H) (p > 0.05 in all field types tested). These results again confirm that place fields on the photostimulation area in both the pre-run and opto run are more stably maintained in a subsequent run period. Similar to Figures S4B and 4C, to compensate for the effect of photostimulation-induced changes in in-field firing rates, we randomly downsampled spikes in the opto run or post-run period for each neuron with place fields on the photostimulation area in both the pre-run and opto run so that their average spike rates in the run periods were normalized by a fold change of in-field spike rates between the pre-run and opto run (Figure S4A). The downsampled spike patterns similarly showed significantly higher correlations than those in the no-stimulation control groups (Figure S4D; *Z* = 2.65, p = 0.0081, Mann-Whitney U test). This result confirms that changes in photostimulation-induced excitability alone cannot account for the increased stability of the place fields.

In random phase stimulation, we found that neurons with place fields on the photostimulation area in both the pre-run and opto run showed spatial correlations that were not significantly different from 0 (Figure 5I; *Z* = 1.53, p = 0.13, Mann-Whitney U test versus 0), while the other place field groups from random phase stimulation showed significantly positive correlations (p < 0.05, Mann-Whitney U test versus 0), similar to PP stimulation. The same non-significant result from random phase stimulation was also observed from their downsampled datasets (Figure S4E; *Z* = 0.57, p = 0.57, Mann-Whitney U test versus 0). These results mean that place fields that were present before random phase stimulation specifically became more unstable after random phase stimulation, which is in contrast to the stability of place fields observed from PP stimulation.

All the recordings described above were performed from a familiar linear track, demonstrating pronounced changes in spatial representations by photostimulation. On the other hand, place cells also undergo plastic changes by novel experiences. To examine whether and how much the plasticity and stability of place fields differ between our photostimulation and novel experiences, we additionally recorded from different four rats that performed the same linear track run in a novel room (termed a novel run; n = 136 cells from 4 recording days from 4 rats) between familiar linear track runs (Figure S7A). By depicting joint plots, we evaluated the effects of our poststimulation or experiencing a novel run on changes in place fields across the pre-run and post-run (Figures S7B–S7D). Compared with the no-stimulation control (Figure S7E), both PP stimulation and a novel run showed no significant differences in the proportions of place fields in the pre-run that were not observed in the post-run (Figure S7F; PP stim: χ^2^ = 0.16, p > 0.99; novel run: χ^2^ = 0.27, p > 0.99, chi-squared test followed by Bonferroni correction) and the proportions of place fields that were newly observed in the post-run (Figure S7G; PP stim: χ^2^ = 1.10, p > 0.99; novel run: χ^2^ = 0.38, p > 0.99, chi-squared test followed by Bonferroni correction). In addition, these proportions were not significantly different between the two conditions (χ^2^ = 0.32, p > 0.99, chi-squared test followed by Bonferroni correction). On the other hand, the proportions of place fields that were not observed in the post-run were significantly larger after random phase stimulation (Figure S7F; χ^2^ = 26.75, p = 1.6 × 10^−6^, chi-squared test followed by Bonferroni correction). Compared with the no-stimulation control, the proportions of stable place fields in the post-run were significantly smaller after the novel run and random phase stimulation, but not PP stimulation (Figure S7H; PP stim in total: χ^2^ = 2.47, p = 0.35; random stim in total: χ^2^ = 14.03, p = 1.8 × 10^−4^; novel run: χ^2^ = 8.60, p = 0.010, chi-squared test followed by Bonferroni correction). No significant differences in these proportions were found between the novel run and random phase stimulation (χ^2^ = 0.23, p = 0.63). Taken together, these results demonstrate that experiencing a novel run reduces the stability of place fields to a level comparable to that observed from random phase stimulation.

## Discussion

In this study, we developed an experimental technique in which ChR2-expressing hippocampal cells were photostimulated with a temporal structure replicating phase precession relative to extracellular theta oscillations. This method specifically manipulated theta-related spike patterns without affecting overall theta power. Hippocampal neuronal ensembles showing place fields during PP stimulation, rather than random phase stimulation, showed stronger reactivation during SWRs in rest periods. Furthermore, the stability of these place fields was increased, compared with that of the other place fields outside the photostimulation area.

Theta phase precession of place cells^3^^,^^4^^,^^5^ are unique mechanisms that enable the formation of temporally organized neuronal sequences. Experimental strategies with physical lesions and pharmacological or optogenetic manipulations of the medial septum have been widely utilized to determine causal roles of theta-related neuronal activity in hippocampus-dependent memory and spatial representations.^17^^,^^19^^,^^20^^,^^21^^,^^22^^,^^23^^,^^24^^,^^25^^,^^48^^,^^49^ In addition, subjecting animals to passive movement conditions has also been shown to be effective in reducing these organized theta-related spike patterns.^26^^,^^50^ The majority of these experimental manipulations not only disrupt theta entrainment of neuronal spikes but also weaken entire theta oscillatory power in the hippocampus. Our closed-loop system selectively manipulated theta phase precession-related neuronal activity while preserving overall field theta power in extracellular signals, allowing us to assess pure physiological effects of theta-related neuronal spikes.

An early paper demonstrated an increased stability of place fields by optogenetic excitation with a 1-s continuous photostimulation.^51^ Our photostimulation protocol had a much sparser temporal pattern; each pulse had a duration of 5 ms, and the number of pulses per lap was 6.3 ± 2.1. We demonstrated that such short pulses were sufficient to affect spatial representations, especially when stimulation timing was precisely coordinated with the theta phase. On the other hand, place fields suppressed by optogenetic inhibition have been shown to become unstable in subsequent recordings, highlighting the activity-dependent maintenance of hippocampal spatial maps.^52^ Consistently, our results demonstrated increased stability in response to optogenetic excitation. In addition, we observed increased post-experience SWR-triggered synchronous spikes of neuronal ensembles after PP stimulation, which are a crucial neuronal mechanism for memory consolidation.^27^^,^^28^^,^^29^^,^^30^ As post-experience neuronal reactivation has been shown to be necessary for the maintenance of stable place fields;^31^^,^^32^ the increased stability of spatial maps after optogenetic PP stimulation during an exploration may be explained by enhanced offline neuronal spikes.

Early studies have shown that electrical stimulation locked to a specific theta phase (e.g., trough or peak) exerts different plastic changes in hippocampal synaptic transmission than stimulation corresponding to other phases.^11^^,^^12^^,^^13^^,^^14^^,^^15^ Recent studies have proposed crucial roles of naturally occurring theta phase precession and theta sequence in hippocampal neurons in various functions, including novel learning, memory acquisition and consolidation, nonspatial information processing, and planning of upcoming behavior.^19^^,^^26^^,^^53^^,^^54^^,^^55^^,^^56^^,^^57^^,^^58^^,^^59^ Our observations add to the growing body of evidence indicating that the inherent property of theta-specific plasticity is related to hippocampal neuronal spike patterns underlying memory formation and consolidation.

### Limitations of the study

In both living animals and slice preparations, theta-paced neuronal activation has been shown to be effective in triggering long-lasting changes in hippocampal neurons.^6^^,^^7^^,^^8^^,^^9^^,^^10^^,^^11^^,^^12^^,^^13^^,^^14^^,^^15^^,^^60^ The effects of theta-entrained stimulation on place cell activity observed in our study are possibly mediated by these synapse-level mechanisms. Early studies have reported that optogenetic stimulation induces place field reorganization even in neurons with no photostimulation-induced spikes.^51^^,^^52^ These phenomena may be explained by changes in recurrent excitation and/or feedback inhibition that are coordinately mediated by both stimulated and nonstimulated neuronal ensembles.^42^^,^^51^ In addition, neuronal membrane potential oscillations have been shown to undergo location-specific subthreshold depolarization (i.e., subthreshold field^34^^,^^41^^,^^61^^,^^62^), which correspondingly show phase precession relative to extracellular field theta rhythms.^34^^,^^35^ Considering the observations that certain subthreshold depolarization is sufficient to alter spatial representations in single neurons,^36^^,^^37^ photostimulation-induced subthreshold membrane voltage changes may potentially contribute to the reorganization of the hippocampal circuit. This study with our spike recordings could not detect such subthreshold neuronal activity.

## STAR★Methods

### Key resources table

**Table:** 

REAGENT or RESOURCE	SOURCE	IDENTIFIER
**Chemicals, peptides, and recombinant proteins**		

Isoflurane	Zoetis	Cat#6073253
Praformaldehyde	Nacalai tesque	Cat#M8E4590
Cresyl violet	Sigma-Aldrich	Cat#125K3707
Re-fine Bright	Yamahachi Dental Mfg., Co.	Cat#KH01
Hydrogen Hexachloroplatinate(IV) Hexahydrate	Nacalai tesque	Cat#V8P4386
AAV5-CaMKII-hChR2(H134R)-EYFP	Addgene	#26969
AAVdj-CaMKII-hChR2(H134R)-EYFP	This paper	#26969

**Experimental models:Organisms/strains**		

Sprague Dawley rat	Japan SLC	slc:SD

**Software and algorithms**		

MATLAB R 2022a	MathWorks	https://jp.mathworks.com/
MClust	A.D. Redish	http://redishlab.neuroscience.umn.edu/MClust/MClust.html
Python 2.7	Python Software Foundation	https://www.python.org/about/

**Other**		

Platinum-Iridium tetrode wire	California Fine Wire Company	Cat#CFW0011173
Stimulation electrode	Unique medical	TOG217-049
Microdrive	Custom built	N/A
Micro syringe	Ito	Model: MS-E05
Syringe pump	Muromachi	Model: Legato 100
Cannula	Eicom	Model: AC-5, AG-12, AD-12
Microtome	Leica	Model: SM2010 R
Neural signal processor	BLACKROCK	Model: Cerebus
Single channel pulse generator	WPI	A310

### Resource availability

#### Lead contact

Further information and requests for resources and reagents should be directed to and will be fulfilled by the lead contact, Takuya Sasaki (takuya.sasaki.b4@tohoku.ac.jp).

#### Materials availability

This study did not generate any unique reagents. The CAD files for creating the electrode assembly by 3D printers are available from the lead contact upon request.

### Experimental model and study participant details

#### Animals

All experiments were performed with the approval of the experimental animal ethics committee at the University of Tokyo (approval number: P29-7) and the committee on animal experiments at Tohoku University (approval number: 2022 PhA-004) and according to the NIH guidelines for the care and use of animals.

All Long Evans rats were purchased from SLC (Shizuoka, Japan) and Long Evans rats (3–6 months old) with a preoperative weight of 400–500 g were used in this study. The animals were housed individually and maintained on a 12-h light/12-h dark schedule with lights off at 7:00 a.m. Following at least 1 week of adaptation to the laboratory, the rats were reduced to 85% of their *ad libitum* weight through limited daily feeding. Water was readily available.

### Method details

#### Surgical procedures

The rat underwent surgery to inject virus vectors and implant electrodes and an optical fiber.^63^^,^^64^ The rat was anesthetized with isoflurane gas (0.5–2.5%), and a 2-cm midline incision was made from the area between the eyes to the cerebellum. A craniotomy with a diameter of 1.2–1.6 mm was created above the right dorsal hippocampus (coordinates were described below) using a high-speed drill, and the dura was surgically removed. A glass pipette with a diameter of 30–40 μm was inserted into the hippocampus (at the coordinates described below) and AAV5-CaMKII-hChR2(H134R)-EYFP or AAVdj-CaMKII-hChR2(H134R)-EYFP (7.28 × 10^13^ vg/ml) dissolved in phosphate-buffered saline (PBS; pH 7.4) was injected into the dorsal hippocampal CA1 region at a rate of 100 nL/min for 3 min at the following coordinates: (1) 3.5 mm posterior and 2.4 mm lateral to bregma, 2.4 mm ventral from dura; (2) 3.5 mm posterior and 3.0 mm lateral to bregma, 2.6 mm ventral from dura; (3) 3.5 mm posterior and 3.6 mm lateral to bregma, 2.8 mm ventral from dura. In addition, the virus was injected into the dorsal hippocampal CA3 region at the following coordinates: (1) 3.5 mm posterior and 3.4 mm lateral to bregma, 3.8 mm ventral from dura; (2) 3.5 mm posterior and 3.7 mm lateral to bregma, 4.0 mm ventral from dura; (3) 3.5 mm posterior and 4.0 mm lateral to bregma, 4.0 mm ventral from dura; (4) 3.5 mm posterior and 4.3 mm lateral to bregma, 4.0 mm ventral from dura. After the injection, the injection pipette was left in place for 1 min and then raised 100 μm and again left in place for 5 min. The skin was sutured and anesthesia was discontinued, and the rat was allowed to awaken spontaneously. Following surgery, each rat was housed individually in transparent Plexiglass with free access to water and food.

After 2–4 weeks of the injection, the rat was anesthetized with isoflurane gas (0.5–2.5%), and a 2-cm midline incision was made from the area between the eyes to the cerebellum. Two stainless-steel screws were implanted in the bone above the prefrontal cortex to serve as ground electrodes. Through the craniotomy (3.5 mm posterior and 3.0 mm lateral to bregma), an electrode assembly consisting of 13–15 independently movable tetrodes and an independently movable optical fiber with a diameter of 200 μm located at the center of the tetrodes was stereotaxically implanted. The tips of the tetrode bundles were lowered to the cortical surface, and the tetrodes were inserted 1.0 mm into the brain at the end of the surgery. The tetrodes were constructed from 17-μm-wide polyimide-coated platinum-iridium (90/10%) wire and plated with platinum to reduce their electrode impedances to 150–300 kΩ at 1 kHz. All the recording devices were secured to the skull using stainless-steel screws and dental cement. Following surgery, each rat was housed individually in transparent Plexiglass with free access to water and food for at least 5 days and was then food-deprived until they reached 85% of their previous body weight.

#### Adjusting electrode depth

The rat was connected to the recording equipment via Cereplex M (Blackrock), a digitally programmable amplifier, close to the rat’s head. The output of the headstage was conducted via a lightweight multiwire tether and a commutator to the Cerebus recording system (Blackrock), a data acquisition system. Electrode turning was performed while the rat was resting in a pot placed on a pedestal. Over a period of at least 2 weeks after surgery, tetrode tips were advanced slowly 25–100 μm per day for 14–21 days until spiking cells were encountered in the CA1 layer of the hippocampus, which was identified on the basis of local field potential (LFP) signals and single-unit spike patterns. Once the tetrodes were adjacent to the cell layer, as indicated by the presence of low-amplitude multiunit activity, tetrodes were settled into the cell layer for stable recordings over a period of several days.

#### Linear track run

After surgery, the rat was trained daily for the linear track run in which the rat run back and forth on a linear track (200 × 10 cm with small sides rising 2 cm above the surface of the arm, 70 cm elevated from the floor) to obtain a constant amount of ∼0.2 mL of chocolate milk reward placed at the track end during a 15-min session. In several training days, the recording headstage and cable were attached to the rat’s head so that the rat could become familiar with the recording condition. All behavioral experiments occurred in the dark phase. This training was repeated daily for 15 min until the rat consumed the reward at least 30 times within the 15-min session. To achieve this criterion performance, training lasted at least 7 days. The rat was maintained in a rest box (43 × 25 cm) outside the field for 60 min before and after the run. To monitor the rat’s moment-to-moment position, a red LED was attached to the animal’s neck, and the LED signal position was tracked at 25 Hz using a video camera located on the ceiling and sampled by a laptop computer.

#### Electrophysiological recording and optical stimulation on a recording day

Electrophysiological data collection commenced after stable well-separated unit activity was identified in the hippocampus and the rat reached the criterion performance. LFP recordings were sampled at 2 kHz and lowpass filtered at 500 Hz. Unit activity was amplified and highpass filtered at 750 Hz. Spike waveforms above a trigger threshold (50 μV) were timestamped and recorded at 30 kHz for 1.6 ms.

On a recording day, the rat first stayed in the rest box for 1 h (pre-rest 1) and then performed a series of three run sessions: (1) a 15-min pre-run session, (2) a 15-min opto run session, and (3) a 15-min post-run session. After performing each session, the animal was allowed to rest for 1 h, termed pre-rest 2, post-rest 1, post-rest 2 sessions, during which the floor of the field was cleaned with water and 70% ethanol. Finally, after post-rest 2 session, photostimulation was repeatedly applied to define neurons that directly emitted spikes in response to photostimulation, termed a probe rest session.

The behavioral paradigm of the pre-run session was identical to that of the training. In the opto run session, closed-loop photostimulation with blue light laser pulses (λ = 488 nm; 3–6 mW) was applied to the rat’s hippocampus, as follows by the stimulation protocols described below, when the rat ran from left to right and crossed an area of 0.6–1.6 m (opto direction). No stimulation was applied when the rat ran in the opposite direction (no-opto direction). In the post-run session, photostimulation was removed so that the experimental conditions were similar to those of the pre-run session. The rat then stayed in the rest box for 1 h (post-rest 2). In the probe rest session after the post-rest 2 session, photostimulation of 100–1000-ms blue light laser pulses was delivered to the hippocampus with an interval of 2 s one hundred times (in total, 200 s). Recordings were conducted for at least 2 days.

#### Control linear track run

All recording conditions except the absence of photostimulation were similar to those described above. The rat first stayed in the rest box for 1 h and then performed a series of three run sessions without applying poststimulation, termed run 1, run 2, and run 3 (Figure S7A), each followed by rest sessions, during which the floor of the field was cleaned with water and 70% ethanol.

#### Novel linear track run

All recording conditions except the absence of photostimulation and a novel run as the second run session were similar to those described above. The rat first stayed in the rest box for 1 h and then performed a series of three run sessions: (1) a 15-min pre-run session, (2) a novel run session without applying poststimulation, and (3) a 15-min post-run session (Figure S7A), each followed by rest sessions, during which the floor of the field was cleaned with water and 70% ethanol. In the novel run, the rat was transferred to a different novel room and performed the same run on the same linear track that was also transferred from the familiar room. In this condition, the rat ran well from initial laps.

#### Closed-loop photostimulation

Upon the online computation of theta phase in an LFP signal, closed-loop electrical stimulation was performed using extension code implemented on the Cerebus recording system (Blackrock) and custom-created C code. The digitized signals of the rat locations at individual frames were transferred through the Arduino Uno board (Arduino) to the Cerebus system. When the rat ran from left to right and crossed an area of 0.6–1.6 m (photostimulation area), the following computations were performed. An electrode channel showing a similar LFP theta phase to that observed from the electrode that was closest to the optical fiber was chosen. LFP signals at 30 kHz were bandpass-filtered at 7–8 Hz, and the peak and trough of each theta cycle were estimated in real time. In a phase precession stimulation (PP stim) protocol, the first photostimulation with a 5-ms blue light laser pulse (λ = 488 nm; 3–6 mW) was applied at the time giving the peak of the first theta cycle after the rat entered the photostimulation area. The timing for the next photostimulation (in ms) was computed online at time *t,* giving the *N*-th peak of theta cycles as follows:

$${Interval}_{N}\times {\left(1–Pos\left(t\right)\right)}^{\mu }\text{,}$$

where *Interval*_*N*_ is the estimated time interval between the *n*-th peak and *(n+1)*-th peak, computed based on the previous *n* theta cycles observed in the photostimulation area. When *N* = 1, *Interval*_*1*_ was set at 120 ms (a default value). The rat’s position in the photostimulation area was normalized so that the start point (0.6 m), and the endpoint (1.6 m) were 0 and 1, respectively, and *Pos(t)* was the normalized rat’s position at time *t*. The nonlinear precession index, μ, was set to 0.9.^65^ When the next theta peak unexpectedly comes before estimated time of the next stimulation, the stimulation was skipped. This skipping occasionally happened especially for the first theta cycle immediately after the rats entered into the photostimulation area. Stimulation was terminated when the rat left the endpoint of the photostimulation area or moved in the reverse direction. When the rat’s position was not correctly digitized, only one photostimulation was applied in the photostimulation area and data were discarded from the analysis. The distribution of inter-stimulation intervals from all rats is shown in Figure 2G.

The random phase stimulation protocol was applied on different days after the PP stimulation protocol was implemented. For each rat, the average duration per lap in which the rat stayed in the photostimulation area (*Dur*_*photo*_) and the number of photostimulations per lap (*N*_*photo*_) were computed based on the results from the PP stim protocol. When the rat traversed through the photostimulation area, by setting a frame rate to 30 kHz, the probability of administering photostimulation at each frame was determined by *N*_*photo*_/*Dur*_*photo*_.

#### Histological analysis to confirm electrode placement

After the experiments, the rats received an overdose of urethane and were intracardially perfused with 4% paraformaldehyde (PFA) in PBS and decapitated. To aid in the reconstruction of the electrode tracks, the electrodes were not withdrawn from the brains until more than 3–4 h after perfusion. After dissection, the brains were fixed overnight in 4% PFA and then equilibrated with a sequence of 20% sucrose and 30% sucrose in PBS. Frozen coronal slices (50 μm) were cut using a microtome, and serial sections were mounted and stained with 4′-6-diamidino-2-phenylindole (DAPI). EYFP expression was confirmed under a fluorescent microscopy. The positions of all the tetrodes were confirmed by identifying the corresponding electrode tracks in histological tissue with an optical microscope (All-in-One Fluorescence Microscope BZ-X710, Keyence Corporation, Osaka, Japan). To preserve the slices and electrode tracks, they were rinsed in water, counterstained with cresyl violet, and coverslipped with hydrophobic mounting medium.

### Quantification and statistical analysis

#### Spike sorting

Spike sorting was performed offline using the graphical cluster-cutting software MClust. Rest recordings before and after the behavioral paradigms were included in the analysis to assure recording stability throughout the experiment and to identify hippocampal cells that were silent during behavior. Clustering was performed manually in 2D projections of the multidimensional parameter space (i.e., comparisons between waveform amplitudes, the peak-to-trough amplitude differences, and waveform energies, each measured on the four channels of each tetrode). The cluster quality was measured by computing the L_ratio_ and isolation distance.^66^ The L_ratio_ was computed by the original equation, proposed by Schmitzer-Torbert et al. (2005), not normalized by the total number of spikes recorded on the tetrode. A cluster was considered as a cell when the L_ratio_ was less than 0.38 (average L_ratio_ was 0.100 ± 0.005 with a median of 0.085 in 332 isolated cells with photostimulation) and the isolation distance was more than 13 (average isolation distance was 35.5 ± 2.8 with a median of 23.1) (Figure S2A). In the auto-correlation histograms, cells with no clear refractory period (<3 ms) were excluded from analyses. Refractory periods of spikes were considered to increase confidence in the successful isolation of cells. In addition, in the cross-correlation histograms, putative cell pairs with a symmetrical gap around the center bins were considered to arise from the same cell and were merged. For each cell, average firing rate over an entire recording period and time difference between the time giving the peak and trough in averaged spike waveform were computed. By applying Gaussian mixture model (GMM) to these two variables, putative excitatory and inhibitory cells were defined as a cell group with lower and higher firing rates, respectively. Cells with an average firing rate of more than 3 Hz were considered to be putative inhibitory cells (n = 10 cells) and were excluded from all analyses, unless otherwise specified.

#### Analysis of behavioral patterns

The instantaneous speed of each animal’s movement at each frame was computed by the first-order adaptive windowing (FOAW) velocity estimation based on 16 frames (640 ms) before the target frame. The linear track was divided into two parts: the consummatory area, a region within 25 cm from both track ends, and the running area, a track area outside the consummatory area. A lap was judged as an “error lap” if (i) after departing a consummatory area, the rat returned to the same consummatory area before reaching the opposite consummatory area, or (ii) when all instantaneous speeds at each frame in a lap were ranked, the top 85% included negative speeds (running in the reverse direction). The other laps were judged as successful laps.

#### Analysis of spatial spike patterns

The following analyses were applied for each cell. Only spikes and locations in successful laps were used for analyses. The average firing-rate distribution on each trajectory (“left to right” or “right to left”) in each run session was separately computed along the track by dividing the total number of spikes in each location bin (10 cm) by the total time that the rat spent in that bin. All firing-rate distributions were smoothed by a one-dimensional convolution with a Gaussian kernel with a standard deviation of one pixel (10 cm). A place cell in a run session was defined as a cell based on the following two criteria: (1) the average firing-rate distribution on a trajectory in a session had a maximum firing rate of more than 0.5 Hz, and (2) the maximum firing rate exceeded 2 SDs above the mean, where the SD and the mean were computed from the series of firing rates except the maximum firing rate in that distribution. For each place cell, the place field center was defined as the position with the maximum firing rate in the distribution. Under this criterion, some place cells had one place field in either one of two trajectories, whereas the others had two place fields in both trajectories. The other cells that did not meet the criteria were classified as nonplace cells.

To evaluate the effects of photostimulation on the alteration of place fields, the numbers of place fields that were observed in the photostimulation area in the pre-run but not observed in the track in the opto run or place fields that were not observed in the track in the pre-run but newly observed in the photostimulation area in the opto run were counted. The numbers of fields were normalized by the expected numbers of fields at the corresponding area (e.g. photostimulation area is 1 m) that were computed from the number of all fields observed in the track in the corresponding length in the pre-run and opto run, respectively.

For categorization, neurons with (1) place fields on the photostimulation area in the opto-run only, (2) place fields on the photostimulation area in both the pre-run and opto-run, (3) place fields on the photostimulation area in the pre-run only, were defined. Place fields that did not meet these criteria were classified as “other”. In all types of fields, place fields that shifted their field centers by a distance of less than 30 cm across sessions were classified as “stable”.

For each cell with a place field at each direction, an information density was computed by the following formula:

$$I=\sum_{i=1}^{N}p_{i}\frac{r_{i}}{r}\log_{2}\frac{r_{i}}{r}$$

where *I* is the information density measured in bits per spike, *i* is the index of the pixels of the place field, *p*_*i*_ is the probability of the animal being at location *i*, *r*_*i*_ is the average firing rate of the cell when the animal is at location *i*, and *r* is the total average firing rate.

For a place cell with place fields on the photostimulation area in both the pre-run and opto run, the ratio of an in-field firing rate in the photostimulation area in the opto run (PF_2_) to that in the pre-run (PF_1_) was computed, which was considered to represent the effect of photostimulation-induced change in cell’s excitability (Figure S4). When the ratio was more than 1, the spike count was randomly downsampled in the post-rest 1 period or the post-run period until the average firing rate became its PF_1_/PF_2_ times. When the ratio was less than 1, the spike count was randomly downsampled in the pre-rest 2 period or the opto-run period until the average firing rate became their PF_2_/PF_1_ times. Changes in SWR-triggered reactivation and spatial correlations were computed from a downsampled data. This analysis was repeated 100 times and their averages were computed as a compensated value obtained from downsampled datasets.

To measure the degree to which a given cell pair exhibited synchronous spikes, termed cofiring,^46^^,^^47^ the numbers of spikes were counted in consecutive 50-ms windows in each of the two cells, creating N-dimensional vectors *x* and *y*, where N is the total number of windows. Pearson’s correlation coefficients were computed between the two vectors as follows:

$$Cofiring=\frac{\sum_{i=1}^{N}\left(x_{i}-\bar{x}\right)\left(y_{i}-\bar{y}\right)}{\sqrt{{\sum_{i=1}^{N}\left(x_{i}-\bar{x}\right)}^{2}}\sqrt{{\sum_{i=1}^{N}\left(y_{i}-\bar{y}\right)}^{2}}}$$

This analysis was applied to all possible cell pairs in which both of the cells showed spike rates of more than 0.2 Hz. If one cell in a cell pair had a spike rate of 0 Hz whereas the other cell had a spike rate of >0.2 Hz, cofiring was computed as 0.

#### LFP analysis

To compute the time-frequency representation of LFP power, an LFP signal was convolved using complex Morlet wavelet transformation. The absolute power spectrum of the LFP during each 10-ms time window was calculated, and power was normalized by average power during running on the photostimulation area with PP stimulation.

To detect SWRs, an LFP signal was bandpass filtered at 150–250 Hz, and the root-mean-square power was calculated in the ripple-band with a bin size of 10 ms and a sliding window of 0.5 ms. SWR events were detected if the power exceeded a threshold for at least 15 ms. The threshold for SWR detection was set to 3 standard deviations (SDs) above the mean of all envelopes.

#### Phase precession analysis

An LFP signal was bandpass filtered at 5–10 Hz. The quality of phase precession within a place field was defined as the circular linear correlation coefficient (CLCC) between spike phases and the positions of animals.^18^ First, spike patterns in the photostimulation area from when the rats run toward the opto direction were specifically extracted. As the average number of stimulation applied in both photostimulation protocols was approximately 6, we analyzed spikes observed within six theta cycles after the rats first entered into the photostimulation area. This time period was almost equivalent to the duration in which the rats almost passed through the photostimulation area with sufficient running speed and clear phase precession was observed. Cells with at least 5 spikes in the photostimulation area were analyzed. If a neuron starts to generate place-selective spikes in its place field before the mice entered into the photostimulation area, their phase precession may start earlier before the area, meaning that the phases of initial parts of spikes might be already advanced. To compensate this effect, we added a slight phase residual (from 0 to π/6 by π/18) to the position-phase plot and computed CLCC for each residual. A true CLCC value was defined as the minimum CLCC among these computations. A significant CLCC was determined when the *p* value of a correlation coefficient was less than 0.01. While the residual in the majority of cells was 0 (no need of the compensation), a minority of cells showed better CLCC with this compensation.

#### Probe test to define neurons that respond to photostimulation

Instantaneous spike rates in the probe test were analyzed to define photoresponsive cell types. A photoexcited cell was defined as a cell based on the following two criteria: (1) average spike probability during 0–100 ms after the onset of photostimulation was more than 10%, and (2) the probability was larger than 5% of the maximum of those computed from 100 surrogate datasets in which spike timing was randomized across time. On the other hand, a photoinhibited cell was defined as a cell based on the following two criteria: (1) the average spike rate without photostimulation was more than 0.2 Hz, and (2) the average spike probability during 0–100 ms was lower than 5% of the minimum of those computed from 100 surrogate datasets in which spike timing was randomized across time. The other cells were classified as nonresponsive cells.

#### Statistical analysis

All data are presented as the mean ± standard error of the mean (SEM), unless otherwise specified, and were analyzed using Python and MATLAB. For each statistical test, data normality was first determined by the F test, and non-parametric tests applied where appropriate. Comparisons of two-sample paired data were analyzed by paired t-test or Signed-rank test. Comparisons of two-sample data were analyzed by Mann–Whitney U test. Multiple group comparisons were performed by post hoc Bonferroni corrections. Comparison of ratio distributions of place field stability between two groups were assessed using Chi-square test. Two-tailed tests were used unless otherwise specified. The null hypothesis was rejected at the p < 0.05 level.

## Data Availability

• The original data will be made available by the lead contact upon request. • Custom MATLAB code will be made available by the lead contact upon request. • Any additional information required to reanalyze the data reported in this work paper is available from the lead contact upon request.
