# Supplementary material for: Effects of theta phase precessing optogenetic intervention on hippocampal neuronal reactivation and spatial maps
Source: iScience. 2023 Jun 28;26(7):107233. doi: 10.1016/j.isci.2023.107233 (PMC10392074; doi:10.1016/j.isci.2023.107233)
Supplement: Document S1. Figures S1–S7 and Table S1 [file mmc1.pdf]

iScience, Volume *26*

Supplemental information

Effects of theta phase precessing optogenetic intervention on hippocampal neuronal reactivation and spatial maps

Yuki Aoki, Taiki Yokoi, Shota Morikawa, Nahoko Kuga, Yuji Ikegaya, and Takuya Sasaki

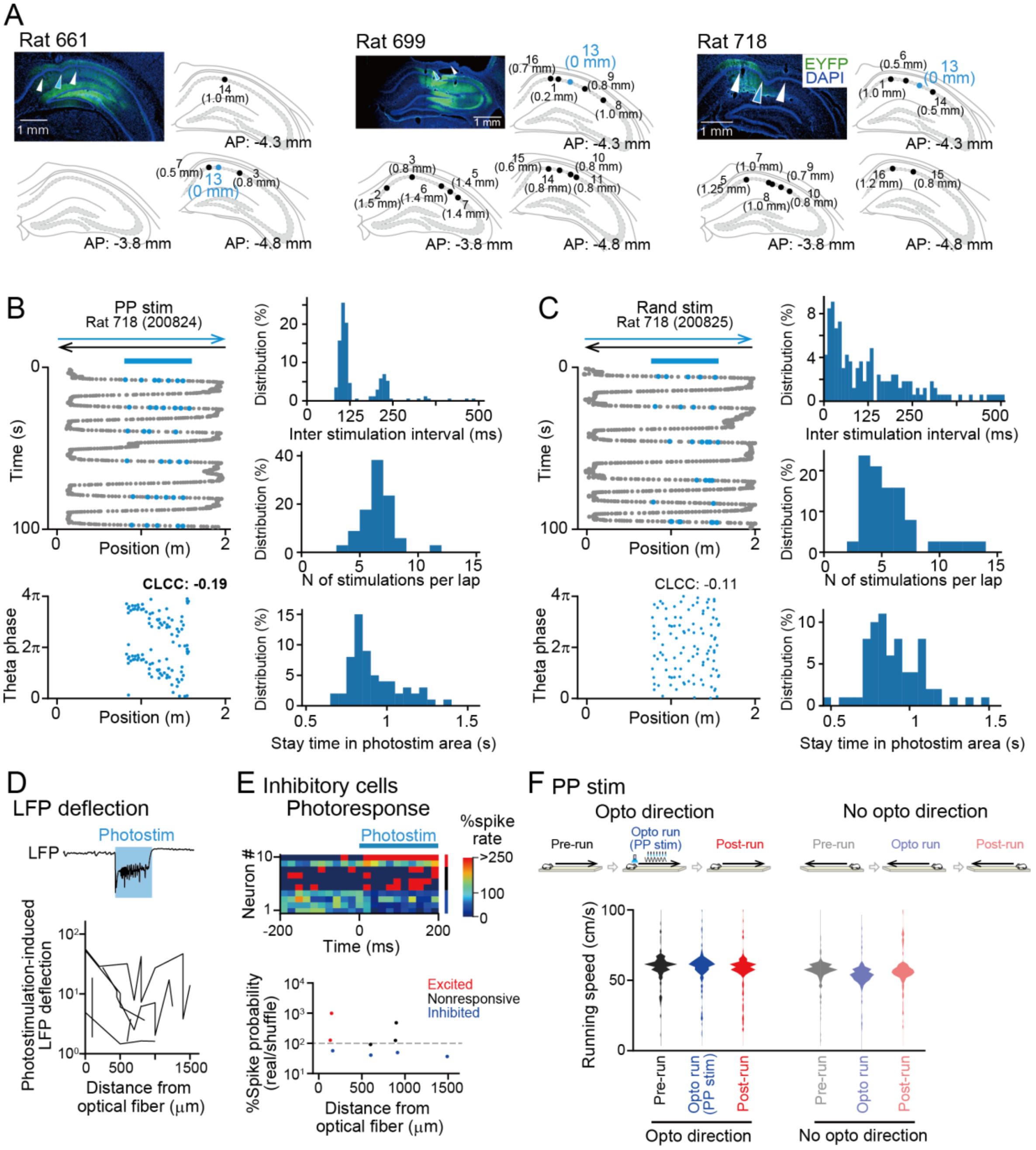

Figure S1. Optogenetic simulation replicating theta phase precession. Related to Figure 1. (A) (Top left) Histological verification of EYFP expression (green) with the locations of the tip of tetrodes (white arrowheads) and an optical fiber (blue arrowhead), superimposed on DAPI-labeled hippocampal neurons (blue). (Bottom and right) The positions of tetrodes (black) and an optical fiber (blue) on the dorsal hippocampal cell layer in sequential coronal brain sections. The length in parentheses indicates distance from the optical fiber. Data from rat 718 are similar to those shown in Figure 1C. (B) A PP stimulation protocol applied to a representative rat is shown. (Top left) Positions of PP stimulation (blue dots) during running

from left to right superimposed on all position samples of trajectories of a rat running back and forth on a linear track (gray dots). (Bottom left) Theta phase of photostimulation (y-axis) represented as a function of position (x-axis). CLCC is shown above (significant CLCC highlighted in bold). (Right) In the rat, distributions of interstimulation intervals within a lap, the number of stimulation per lap, and stay time in the poststimulation area. (C) Same as B but for random phase stimulation. (D) (Top) A typical photostimulation-induced LFP trace in an electrode. (Bottom) Ratios of the amplitude of LFP deflection (averaged absolute amplitude) during 0–100 ms after photostimulation to that during 0–100 ms before photostimulation, plotted as a function of distance from the optical fiber (n = 43 electrodes from 6 rats). For each electrode, a ratio was computed from a representative recording day that included cell units in the electrode. Each line represents each rat. (E) Same as in Figure 1F and 1G but for putative inhibitory cells. (Top) Pseudocolor images showing averaged changes in photostimulation-induced spike rates from all putative inhibitory cells (n = 10 neurons). The time was aligned to the onset of photostimulation, and the neurons were aligned to cell types based on their photoresponsivity. In each neuron, firing rates in individual bins were normalized by average firing rate at 0–200 ms before photostimulation. (Bottom) Ratios of photostimulation-induced spike probabilities in real data to those in shuffled data, plotted as a function of distance from the optical fiber. Each dot represents each neuron. Plots were jittered within 100 μm along the horizontal axis for visualization. Cells that did not exhibit photostimulation-induced spikes were excluded from this graph. (F) Violin plot of running speed (computed at the area of 0.6–1.6 m from each start) in each running direction in each run session. No significant differences were found among these groups revealed by Tukey's test ($P > 0.05$).

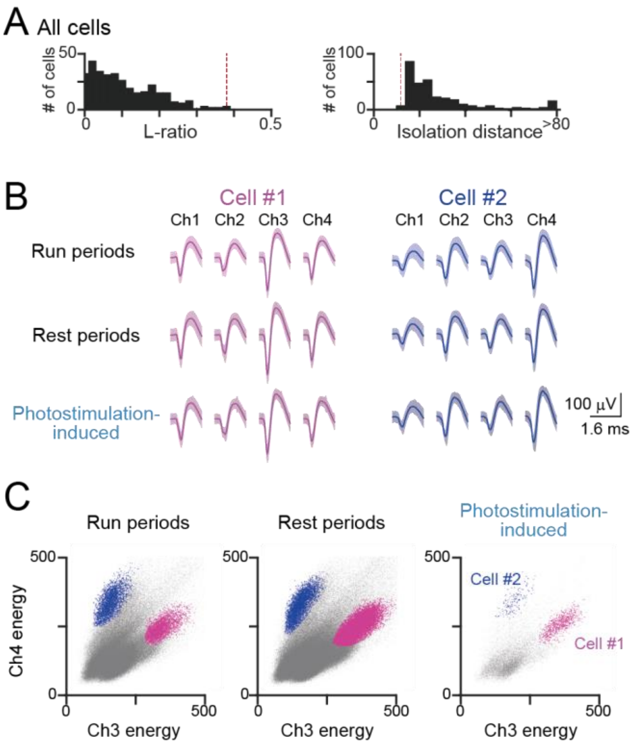

Figure S2. Photostimulation did not affect spike waveforms and cluster quality. Related to Figure 1. (A) Distributions of L_{ratio} and isolation distance for all neuronal units used for analyses (*n* = 332 neurons) identified by multiunit spike sorting. Red lines represent thresholds to define cells. (B) Average spike waveforms of representative two cells labeled in color, corresponding with C. Waveforms are presented as the mean ± SD. (C) Projection patterns of the energy of multiunit signals recorded from two channels in representative single tetrodes. Each panel shows a recording phase (pre-run and photostimulation-related spikes) for the same channel. Each dot represents one spike, and each color represents each cluster that was assigned to a single cell. The patterns of these spike clusters remained stable over the recording periods.

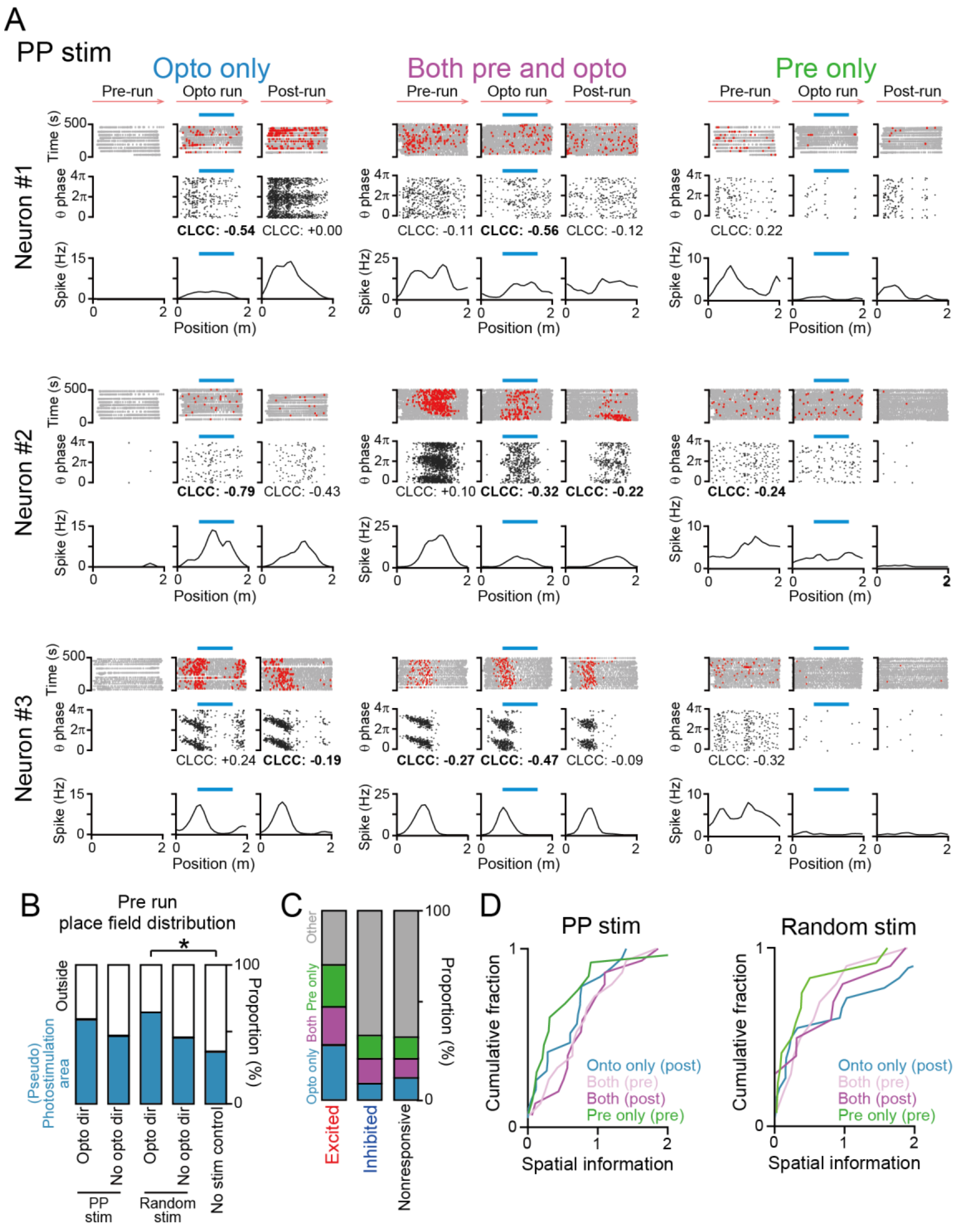

Figure S3. Spatial spike patterns in response to PP stimulation. Related to Figure 3. (A) Typical spatial firing patterns of three neurons with a place field on the photostimulation area in the opto-run only (left), place fields on the photostimulation area in both the pre-run and opto-run (middle), and a place field on the photostimulation area in the pre-run only (right). In each cell, the top panels show trajectories (gray) with locations of individual spikes (red)

in representative laps. The middle panels show theta phase of spikes as a function of animal's position (two theta cycles). CLCC is shown below for each place field defined in a run session (significant CLCC highlighted in bold). The bottom panels show spatial firing rate map. The upper cyan bars represent the photostimulation area. (B) The proportions of place fields observed in the (pseudo) photostimulation area (an area of 0.6–1.6 m from the start) (cyan) and outside the area (black) in each recording condition in the pre-run. The data were constructed from the plots in Fig. 3B–F (opto direction in PP stim, Fig. 3B; no opto direction in PP stim, Fig. 3C; opto direction in random stim, Fig. 3D; no opto direction in random stim, Fig. 3E; no stim control, Fig. 3F). The distributions were significantly different between the opto direction in random stim and the no stim control ($\chi^2 = 9.15$, $P = 0.025$, Chi–square test followed by Bonferroni correction), whereas they were not significantly different across the other pairs of groups ($P > 0.05$, Chi–square test followed by Bonferroni correction). (C) The proportions of photostimulation-related place fields, separately analyzed for cell types based on their photoresponsivity defined in Fig. 1F (n = 45, 50, and 227 cells). (D) Spatial information of individual photostimulation-related place cells in the pre-run and post-run. $P > 0.05$, Mann–Whitney U test among groups.

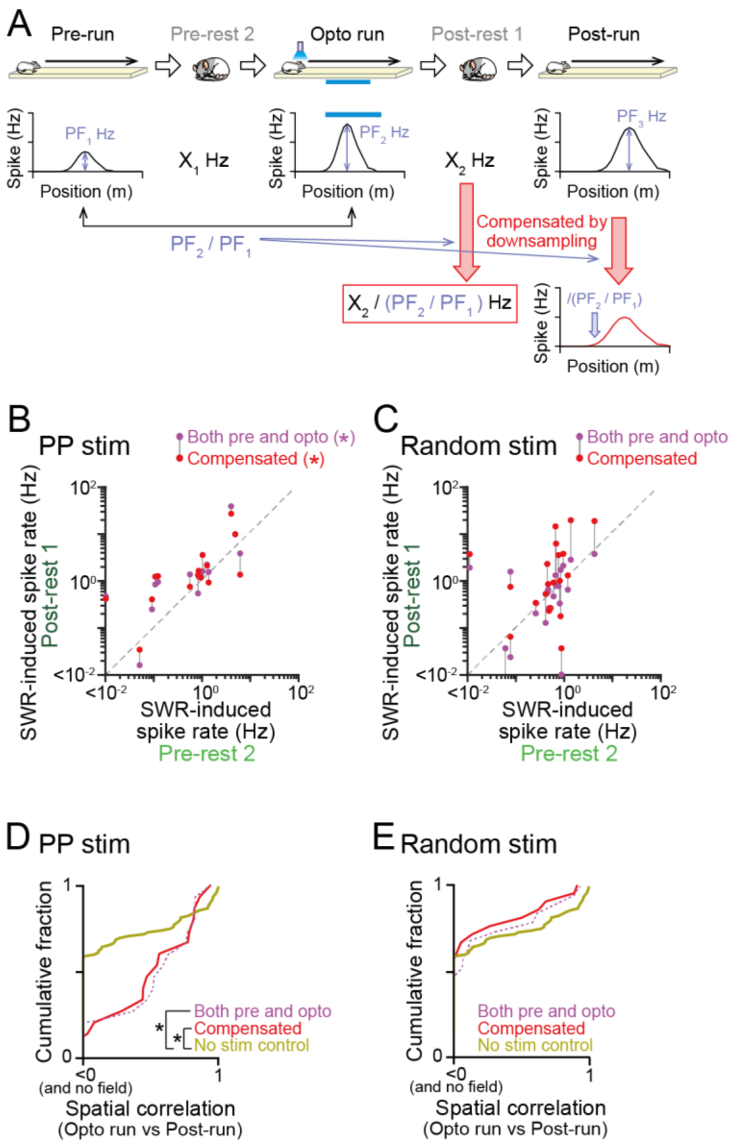

Figure S4. Changes in spike rates in the photostimulation area alone do not account for the increased reactivation and place field stability in cells showing place fields on the photostimulation area. Related to Figure 4 and 5. (A) For a cell showing place fields on the photostimulation area in both the pre-run and opto-run, the ratio of an in-field firing rate in the photostimulation area in the opto run (PF_2) to that in the pre-run (PF_1) was computed. When the ratio was more than 1, spike counts were randomly downsampled in the post-rest 1 and the post-run periods until the average firing rates became their PF_2 / PF_1 times. When the ratio was less than 1, spike counts were randomly downsampled in the pre-rest 2 and the opto-run periods until the average firing rates became their PF_2 / PF_1 times. (B) Similar to Figure 4F, SWR-induced spike rates of individual cells with place fields on the photostimulation area in both the pre-run and opto-run were compared between the pre-rest 2 and post-rest 1 periods (n = 15 cells). For each cell, magenta and red dots represent an

original data and the corresponding downsampled data, connected by a gray line. Both datasets show significantly higher spike rates in post-rest 1 periods, as indicated by * (both pre and opto: $Z = 2.16$, $P = 0.030$; compensated: $Z = 2.16$, $P = 0.030$, Wilcoxon signed-rank test). (C) Same as B but for random phase stimulation (n = 21 cells). No significant differences were found in both datasets (both pre and opto: $Z = 1.89$, $P = 0.059$; compensated: $Z = 2.16$, $P = 0.030$, Wilcoxon signed-rank test). (D) Cumulative distributions of spatial correlations of cells with place fields on the photostimulation area in both the pre-run and opto-run between the opto run and post-run. Correlations from cells with no fields observed in either one of the run sessions were computed as 0. *$P < 0.05$, Mann–Whitney U test. (E) Same as D but for random phase stimulation ($P > 0.05$, Mann–Whitney U test versus 0).

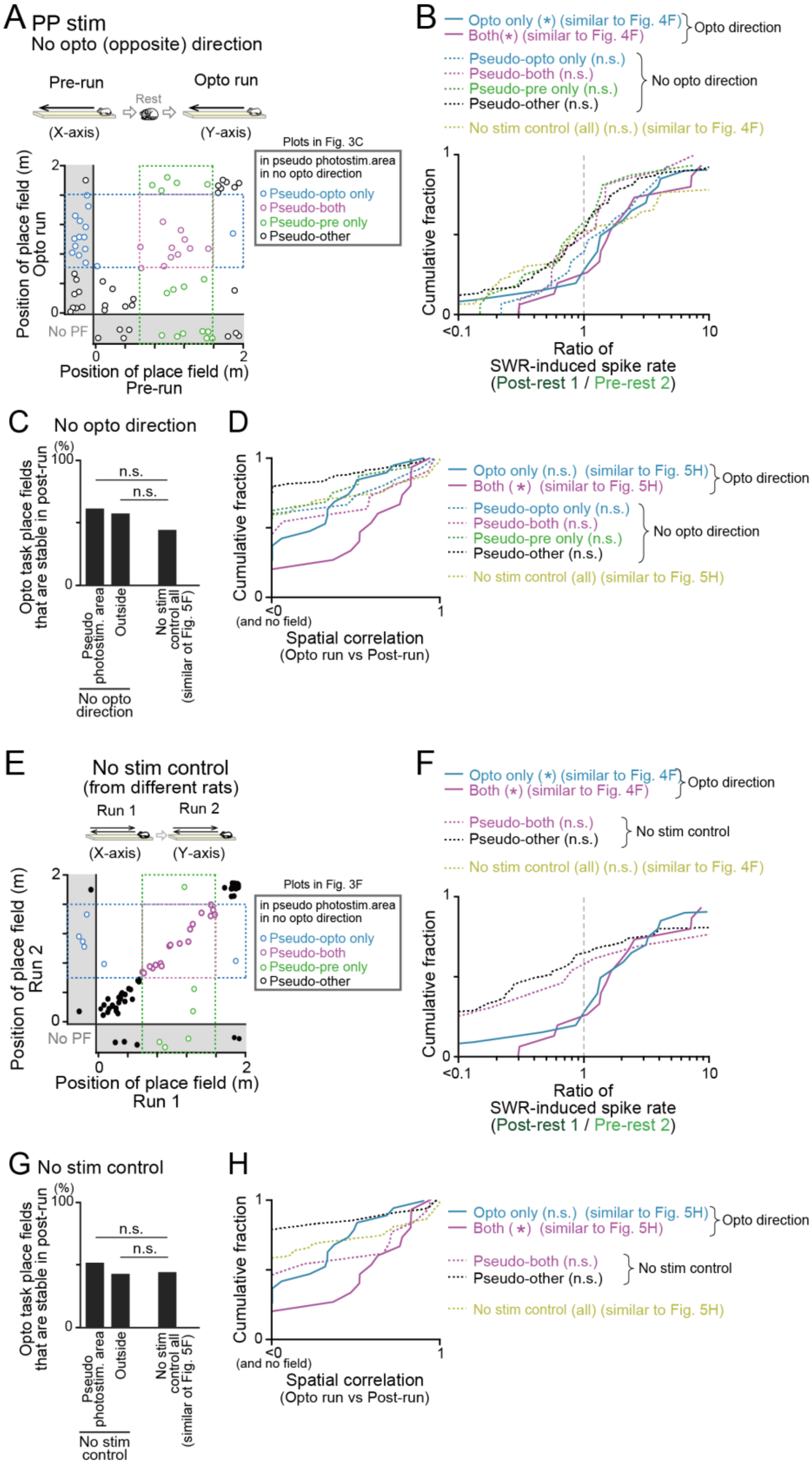
A PP stim
No opto (opposite) direction
Pre-run
Rest
Opto run
(X-axis)
(Y-axis)
Plots in Fig. 3C
in pseudo photostim.area
in no opto direction
Pseudo-opto only
Pseudo-both
Pseudo-pre only
Pseudo-other
Position of place field (m)
Opto run
No PF
Position of place field (m)
Pre-run
B
Opto only (∗) (similar to Fig. 4F)
Both(∗) (similar to Fig. 4F)
Opto direction
Pseudo-opto only (n.s.)
Pseudo-both (n.s.)
Pseudo-pre only (n.s.)
Pseudo-other (n.s.)
No opto direction
No stim control (all) (n.s.) (similar to Fig. 4F)
Cumulative fraction
Ratio of
SWR-induced spike rate
(Post-rest 1 / Pre-rest 2)
C No opto direction
Opto task place fields
that are stable in post-run
(%)
n.s.
n.s.
Pseudo photostim. area
Outside
No opto direction
No stim control all (similar ot Fig. 5F)
D
Opto only (n.s.) (similar to Fig. 5H)
Both (∗) (similar to Fig. 5H)
Opto direction
Pseudo-opto only (n.s.)
Pseudo-both (n.s.)
Pseudo-pre only (n.s.)
Pseudo-other (n.s.)
No opto direction
No stim control (all) (similar to Fig. 5H)
Cumulative fraction
(and no field)
Spatial correlation
(Opto run vs Post-run)
E
No stim control
(from different rats)
Run 1
Run 2
(X-axis)
(Y-axis)
Plots in Fig. 3F
in pseudo photostim.area
in no opto direction
Pseudo-opto only
Pseudo-both
Pseudo-pre only
Pseudo-other
Position of place field (m)
Run 2
No PF
Position of place field (m)
Run 1
F
Opto only (∗) (similar to Fig. 4F)
Both (∗) (similar to Fig. 4F)
Opto direction
Pseudo-both (n.s.)
Pseudo-other (n.s.)
No stim control
No stim control (all) (n.s.) (similar to Fig. 4F)
Cumulative fraction
Ratio of
SWR-induced spike rate
(Post-rest 1 / Pre-rest 2)
G No stim control
Opto task place fields
that are stable in post-run
(%)
n.s.
n.s.
Pseudo photostim. area
Outside
No stim control
No stim control all (similar ot Fig. 5F)
H
Opto only (n.s.) (similar to Fig. 5H)
Both (∗) (similar to Fig. 5H)
Opto direction
Pseudo-both (n.s.)
Pseudo-other (n.s.)
No stim control
No stim control (all) (similar to Fig. 5H)
Cumulative fraction
(and no field)
Spatial correlation
(Opto run vs Post-run)

Figure S5. No significant results from no opto direction and no stimulation control groups. Related to Figure 4 and 5. (A) Joint plots showing changes in the positions of the place field center between the pre-run (x-axis) and the opto run (y-axis) for the opposite (no opto) direction (n = 73 cells that had at least one place field in the two sessions). The data are similar to those shown in Fig. 3C but the plots are labeled in a different color depending on the types of place fields related to the pseudo photostimulation area. (B) Cumulative distributions of percentage changes in SWR-induced spike rates between the post-rest 1 and pre-rest 2 periods for individual cell types defined from the no opto direction (dotted lines; pseudo-opto only; n = 15 cells, Z = 0.97, P = 0.33; pseudo-both; n = 11 cells, Z = 0.18, P = 0.86; pseudo-pre only; n = 16 cells, Z = 0.36, P = 0.71; pseudo-other; n = 104 cells, Z = 0.75, P = 0.45, Wilcoxon signed-rank test). For comparison, the data similar to Fig. 4F are represented as solid lines. * represents a significantly higher spike rate in the post-rest 1 period, compared with the pre-rest 2 period (P < 0.05, Wilcoxon signed-rank test). (C) The proportions of place fields that were stable in the post-run (n = 26 cells, χ^2 = 0.66, P = 0.83; n = 33 cells, χ^2 = 0.44, P > 0.99 0.027, Chi–square test followed by Bonferroni correction). For comparison, the data similar to Figure 5F are presented in the right column. P > 0.05, Chi–square test followed by Bonferroni correction. (D) Cumulative distributions of spatial correlations between the opto run and post-run for individual cell types defined from the no opto direction (dotted lines; pseudo-opto only; n = 15 cells; pseudo-both; n = 11 cells; pseudo-pre only; n = 16 cells; pseudo-other; n = 104 cells; P > 0.05 in all field types tested, Mann–Whitney U test). Correlations from cells with no fields observed in either one of the sessions were computed as 0. For comparison, the data similar to Fig. 5H are represented as solid lines. *P < 0.05, Mann–Whitney U test versus no stimulation control. (E) Joint plots between Run 1 (x-axis) and Run 2 (y-axis) for the no stimulation control group. The data are similar to those shown in Figure 3F (n = 69 fields that were recorded in at least one run) but the plots are labeled in a different color depending on the types of place fields related to the pseudo-photostimulation area. (F) Cumulative distributions of percentage changes in SWR-induced spike rates between post-rest 1 and pre-rest 2 periods for individual cell type defined from the no stimulation control (dotted lines; pseudo-both; n = 13 cells, Z = 1.45, P = 0.14; pseudo-other; n = 49 cells, Z = 1.78, P = 0.074, Wilcoxon signed-rank test). Data from the pseudo-opto only and pseudo-pre only were not presented due to the limitation of the number of samples. For comparison, the similar to Fig. 4F are represented as solid lines. * represents a significantly higher spike rate in the post-rest 1 period, compared with the pre-rest 2 period (P < 0.05, Wilcoxon signed-rank test). (G) The proportions of place fields that were stable in the post-run (n = 25 cells, χ^2 = 0.41, P > 0.99; n = 51 cells, χ^2 = 0.026, P > 0.99, Chi–square test followed by Bonferroni correction). For comparison, the data similar to Fig. 5F are presented in the right column. P > 0.05, Chi–square test followed by Bonferroni correction. (H) Cumulative distributions of spatial correlations between the opto run and post-run for individual cell types defined from the no stimulation control (dotted lines; pseudo-both; n = 13 cells; pseudo-other; n = 49 cells; P > 0.05 in all field types tested, Mann–Whitney U test). Correlations from cells with no fields observed in either one of the sessions were computed as 0. For comparison, the data similar to Fig. 5H are presented as solid lines. *P < 0.05, Mann–Whitney U test versus no stimulation control.

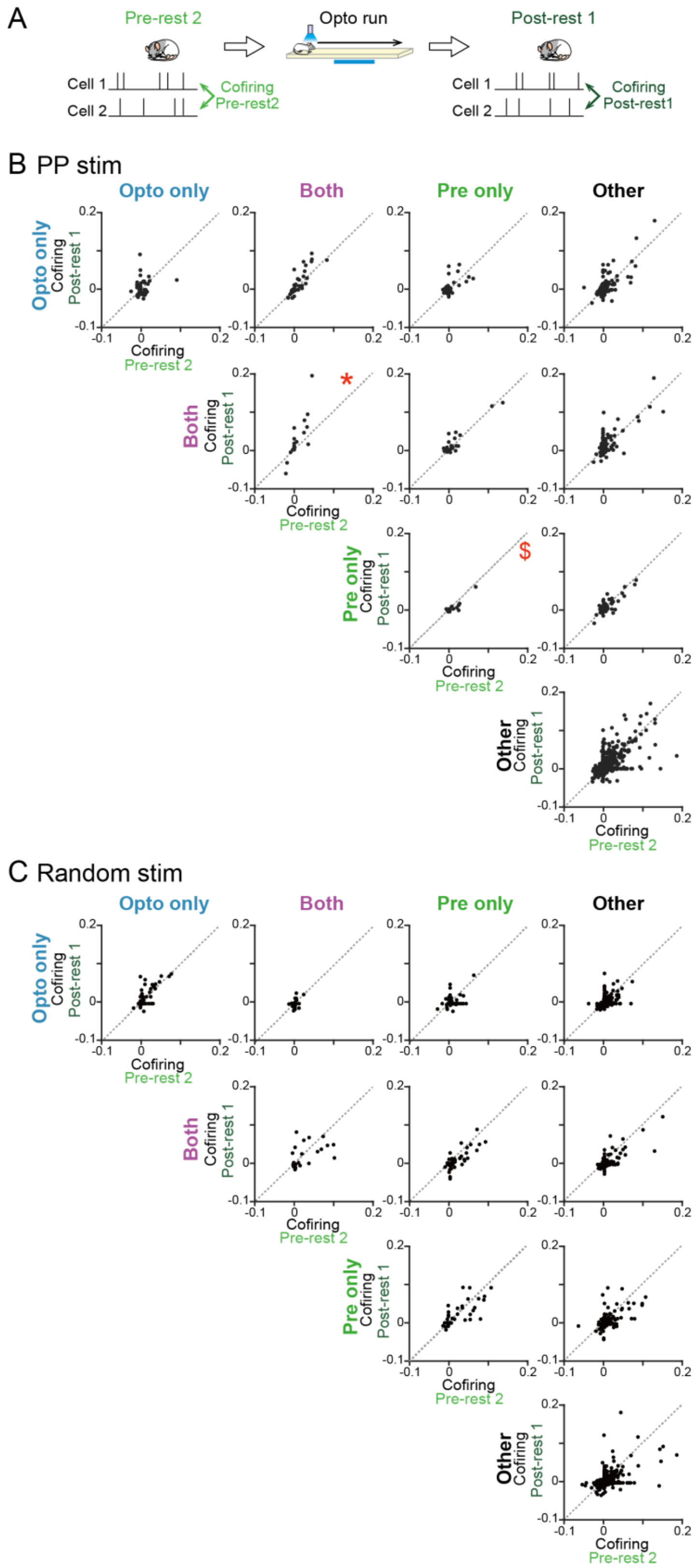
A
Pre-rest 2
Opto run
Post-rest 1
Cell 1
Cell 2
Cofiring
Pre-rest2
Cofiring
Post-rest1
B PP stim
Opto only
Both
Pre only
Other
Cofiring
Post-rest 1
Cofiring
Pre-rest 2
*
$
C Random stim
Opto only
Both
Pre only
Other

Figure S6. Cofiring analysis from all pairs of neuron types. Related to Figure 4. (A) Schematic illustration of computation of cofiring from the two rest periods. (B) Comparison of cofiring of pairs of neurons between the pre-rest 2 and post-rest 1 periods in PP stimulation protocols. Each dot represents each cell pair. For each panel, neuron pairs with a neuron classified into a cell type described above (opto only, both, pre only, or other) and a neuron classified into a cell type described on the left were analyzed. * and $ represent significantly higher and lower cofiring in the post-rest 1 period, compared with the pre-rest 2 period, respectively ($P < 0.05$, Wilcoxon signed-rank test). The graphs from both vs both and pre only vs pre only were also presented in Fig. 4I. (C) Same as B but for random phase stimulation ($P > 0.05$ in all comparisons, Wilcoxon signed-rank test). The graphs from both vs both and pre only vs pre only were also presented in Fig. 4J.

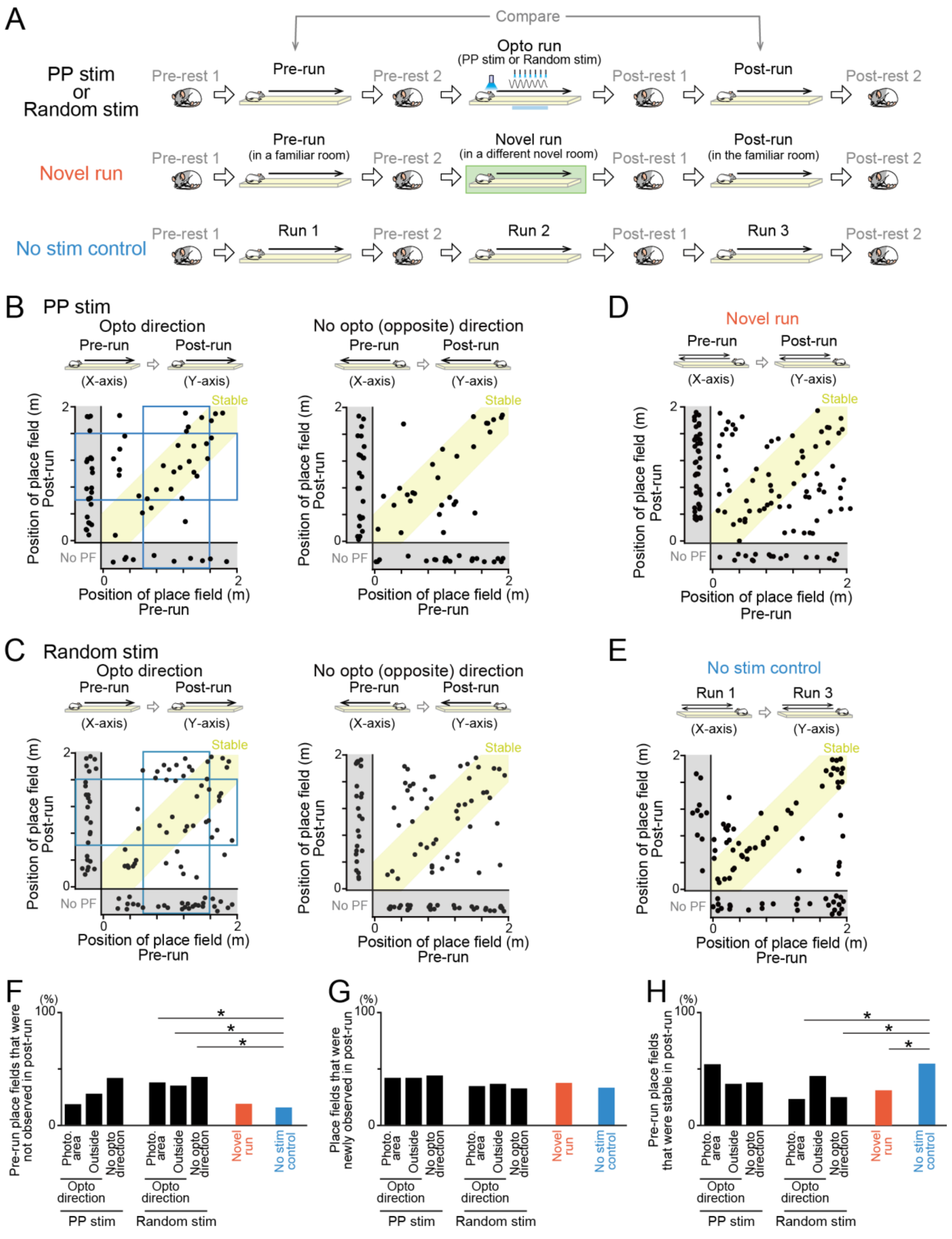
A
Compare
PP stim
or
Random stim
Pre-rest 1
Pre-run
Pre-rest 2
Opto run
(PP stim or Random stim)
Post-rest 1
Post-run
Post-rest 2
Novel run
(in a familiar room)
(in a different novel room)
(in the familiar room)
No stim control
Run 1
Run 2
Run 3
B
PP stim
Opto direction
No opto (opposite) direction
(X-axis)
(Y-axis)
Stable
Position of place field (m)
No PF
C
Random stim
D
E
F
G
H
(%)
Pre-run place fields that were not observed in post-run
Place fields that were newly observed in post-run
Pre-run place fields that were stable in post-run
Photo. area
Outside
No opto direction
Novel run
No stim control
Opto direction
PP stim
Random stim

Figure S7. Results from a novel run. Related to Figure 5. (A) Schematic illustration of recording procedures. On a recording day, a rat performed three 15-min linear track run sessions. The second run session included photostimulation (top; PP stim or random stim), a novel run in a different room shown in right green (middle; novel run), or the same familiar run without any changes (bottom; no stim control). (B–E) Joint plots showing changes in the positions of the place field center between the pre-run (x-axis) and the post-run (y-axis) after the opto-run with PP stimulation (B; n = 67 and 73 cells that had at least one place field in the two run sessions), random phase stimulation (C; n = 109 and 101 cells), a novel run (D; n = 91 cells), and a control run without stimulation (E; n = 134 cells). The yellow region represents stable place fields that shifted their field center with a distance of less than 30 cm across the two sessions. The blue area represents the photostimulation area. Plots were jittered within 5 cm for visualization. (F) (From left to right) The proportions of place fields that were eliminated in the post-run after PP stimulation (three columns, n = 43, 43, and 50 cells in total), random phase stimulation (three colmuns, n = 81, 81, and 86 cells in total), a novel run (n = 90 cells in total), and a control run (n = 64 cells in total). *P < 0.05, Chi–square test followed by Bonferroni correction. (G) The proportions of place fields that were newly observed in the post-run after PP stimulation (three columns, n = 57, 57, and 52 cells in total), random phase stimulation (three columns, n = 80, 80, and 76 cells in total), a novel run (n = 117 cells in total), and a control run (n = 81 cells in total). P > 0.05, Chi–square test followed by Bonferroni correction. (H) The proportions of place fields that were stable in the post-run after PP stimulation (three columns, n = 24, 19, and 50 cells in total), random phase stimulation (three colmuns, n = 42, 35, and 86 cells in total), a novel run (n = 90 cells in total), and a control run (n = 64 cells in total). *P < 0.05, Chi–square test followed by Bonferroni correction.

Rat ID	Experiment	N of putative excitatory cells
Rat 1	PP stimulation Day 1	29
Rat 2	PP stimulation Day 1	37
	PP stimulation Day 2	17
	Random Stimulation Day 1	34
	Random Stimulation Day 2	25
Rat 3	PP stimulation Day 1	27
	PP stimulation Day 2	36
	Random Stimulation Day 1	37
Rat 4	Random Stimulation Day 1	8
	Random Stimulation Day 2	15
	Random Stimulation Day 3	11
Rat 5	Random Stimulation Day 1	11
	Random Stimulation Day 2	20
	Random Stimulation Day 3	12
Rat 6	Random Stimulation Day 1	3
Rat 7	No stim control	6
Rat 8	No stim control	27
Rat 9	No stim control	39
Rat 10	Novel run	24
Rat 11	Novel run	27
Rat 12	Novel run	47
Rat 13	Novel run	38

Table S1. Summary of the number of cells recorded from individual rats. Related to Figure 1. To avoid overlaps of cells sampled across days, cells were collected from different tetrodes in each day for different experiments.
